# Incorporation of Unmanned Aerial Vehicle (UAV) Point Cloud Products into Remote Sensing Evapotranspiration Models

**DOI:** 10.3390/rs12010050

**Published:** 2020

**Authors:** Mahyar Aboutalebi, Alfonso F. Torres-Rua, Mac McKee, William P. Kustas, Hector Nieto, Maria Mar Alsina, Alex White, John H. Prueger, Lynn McKee, Joseph Alfieri, Lawrence Hipps, Calvin Coopmans, Nick Dokoozlian

**Affiliations:** 1 Department of Civil and Environmental Engineering, Utah State University, Logan, UT 84322, USA;; 2 U. S. Department of Agriculture, Agricultural Research Service, Hydrology and Remote Sensing Laboratory, Beltsville, MD 20705, USA;; 3 Complutum Tecnologías de la Información Geográfica (COMPLUTIG), 28801 Madrid, Spain;; 4 E & J Gallo Winery Viticulture Research, Modesto, CA 95354, USA;; 5 Plants, Soils and Climate Department, Utah State University, Logan, UT 84322, USA; 6 Department of Electrical and Computer Engineering, Utah State University, Logan, UT 84322, USA

**Keywords:** point-cloud, TSEB, LAI, evapotranspiration (ET), GRAPEX, AggieAir, UAS, UAV, VSSIXA

## Abstract

In recent years, the deployment of satellites and unmanned aerial vehicles (UAVs) has led to production of enormous amounts of data and to novel data processing and analysis techniques for monitoring crop conditions. One overlooked data source amid these efforts, however, is incorporation of 3D information derived from multi-spectral imagery and photogrammetry algorithms into crop monitoring algorithms. Few studies and algorithms have taken advantage of 3D UAV information in monitoring and assessment of plant conditions. In this study, different aspects of UAV point cloud information for enhancing remote sensing evapotranspiration (ET) models, particularly the Two-Source Energy Balance Model (TSEB), over a commercial vineyard located in California are presented. Toward this end, an innovative algorithm called Vegetation Structural-Spectral Information eXtraction Algorithm (VSSIXA) has been developed. This algorithm is able to accurately estimate height, volume, surface area, and projected surface area of the plant canopy solely based on point cloud information. In addition to biomass information, it can add multi-spectral UAV information to point clouds and provide spectral-structural canopy properties. The biomass information is used to assess its relationship with in situ Leaf Area Index (LAI), which is a crucial input for ET models. In addition, instead of using nominal field values of plant parameters, spatial information of fractional cover, canopy height, and canopy width are input to the TSEB model. Therefore, the two main objectives for incorporating point cloud information into remote sensing ET models for this study are to (1) evaluate the possible improvement in the estimation of LAI and biomass parameters from point cloud information in order to create robust LAI maps at the model resolution and (2) assess the sensitivity of the TSEB model to using average/nominal values versus spatially-distributed canopy fractional cover, height, and width information derived from point cloud data. The proposed algorithm is tested on imagery from the Utah State University AggieAir sUAS Program as part of the ARS-USDA GRAPEX Project (Grape Remote sensing Atmospheric Profile and Evapotranspiration eXperiment) collected since 2014 over multiple vineyards located in California. The results indicate a robust relationship between in situ LAI measurements and estimated biomass parameters from the point cloud data, and improvement in the agreement between TSEB model output of ET with tower measurements when employing LAI and spatially-distributed canopy structure parameters derived from the point cloud data.

## 1. Introduction

Evapotranspiration (ET) is one of the key components in water and energy cycles, and its quantification is essential to increasing crop water use efficiency [1]. However, estimation of ET using physically-based models is not a straightforward process due to input requirements and model complexity [2]. The degree of complexity increases with non-homogeneous landscapes where both soil and vegetation contribute to radiometric temperature and surface energy fluxes [3].

One ET model that has been successful in estimating spatially distributed surface energy fluxes from aerial imagery over different landscapes is the Two-Source Energy Balance model (TSEB) [4]. The TSEB model was developed by Norman et al. [5] to compute surface energy fluxes using a single measurement of remotely-sensed surface temperature (at one view angle) to overcome the difficulties associated with characterizing the impact of canopy structure, fractional cover, sensor view, and sun zenith angle on the radiometric brightness temperature and its relationship to surface aerodynamic temperature. In recent years, numerous studies have evaluated the performance of TSEB-based models at different spatial scales, climates, and landscape heterogeneity.

Satellites and Unmanned Aerial Vehicles (UAVs) offer an opportunity to provide multi-spectral imagery and at different pixel resolutions. Satellites can cover the globe with daily to bi-weekly re-visit times, while UAVs are designed to cover small areas, obtain higher resolution imagery, and capture information at a specific time. One important remote sensing application is estimation of vegetation biomass, and ultimately yield, typically with vegetation indices (VIs), which is easily calculated using multi-spectral imagery. Numerous research studies have been conducted to fit a linear or nonlinear regression model between VIs and biomass parameters [6]. Basically, significant differences in plant reflectances and energy emission in the optical wavelengths, particularly the red (R) and near-infrared (NIR) region, defined as the range between 700 and 1300 nm [7] due to biochemical plant constitutes such as chlorophyll, have resulted in numerous VI formulas [8]. While the performance of VI-based models has been promising, these indices have generally been developed for uniformly distributed canopies, and are thus not as reliable in estimating plant biomass/Leaf Area Index (LAI) for strongly clumped and uniquely structured canopies such as vineyards [9].

A saturation issue occurs with well-developed canopies, wherein, despite significant increases in biomass parameters (and as a result LAI), VI values become saturated, meaning they plateau at a maximum value and are no longer sensitive to increases in LAI [10,11]. Thus, VIs are recommended to be used only in early growing stages in denser canopies [12]. The saturated behavior of VIs versus biomass parameters is more noticeable in normalized VIs, which are set to a specific range (e.g., −1, +1). For example, Diarra et al. [13] evaluated the TSEB model performance using Advanced Spaceborne Thermal Emission and Reflection Radiometer (ASTER) images and the FAO-56 dual crop coefficient approach versus Eddy Covariance records for monitoring actual ET and detecting water stress over irrigated wheat and sugar beets located in the Haouz plain in the center of the Tensift basin (Central Morocco). They concluded that TSEB performed very well, even at a large scale. However, to estimate LAI based on the vegetation indices (VIs), they found that *LAI >* 2.5 saturates the normalized difference vegetation index (NDVI) and no relationship can be found between NDVI and LAI. In contrast, *LAI <* 1.5 resulted in a quite linear relationship between NDVI and LAI. Although LAI is a critical input for ET models, accurate estimation of LAI using only VIs is not possible, particularly when the canopy is well-developed or is uniquely structured. In addition, investigation of the relationship between direct or indirect in situ LAI measurements and VIs is certainly time-consuming and labor-intensive [14]. Thus, exploring new techniques to minimize the need for calibration of remote sensing retrieval of LAI has significant advantages for application in complex canopies.

The development of lightweight UAVs has provided an opportunity for acquiring very high-resolution multi-spectral imagery (less than 50 cm pixel^−1^) to produce ortho-mosaics and 3D information products such as point-cloud and digital surface models (DSMs) using photogrammetry algorithms [15]. UAV imagery has been widely used in agricultural activities and in extensive research in areas such as yield mapping [16], plant heath monitoring [17], plant water status [18], irrigation efficiency [19], phenotyping [20], and weed and pest detection [21,22]. In comparison with satellites, UAVs are cost-effective, easy to operate, and portable, while offering very high-resolution products [23]. In addition to these features, dense 3D dense information can be generated for objects from the overlapping imagery captured by UAVs to be used in mapping plant canopy structure and volume that is likely to be more directly correlated to plant biomass and LAI than VIs.

This 3D source of information from UAV imagery is also called a point cloud, which is a dataset representing visible parts of objects where light is reflected [24]. This source can be produced by three-dimensional point-cloud modeling, photogrammetry, and computer visualization algorithms. Two popular algorithms developed for generating point cloud datasets are Structure from Motion (SfM) and multiview-stereo (MVS), recommended for when optical cameras are used as opposed to expensive laser scanners [15]. Although 3D information for an object can be directly and accurately provided by Light Detection and Ranging (LiDAR) installed on manned and unmanned aerial vehicles, collecting point-cloud information using photogrammetry methods is much less expensive, thus representing an economically viable alternative. In addition, the SfM method requires neither external camera calibration parameters (i.e., position and orientation) nor internal parameters (i.e., lens properties) to perform the bundle adjustment to reconstruct a 3D scene [25]. In some cases, UAV point clouds provide more details of small objects than airborne LiDAR datasets. For instance, the authors in [26] found that 45 out of 205 trees were not detected when they used an airborne LiDAR dataset, while only 14 trees were missed using a UAV photograph-based point cloud. Compared to LiDAR technology, the main weakness of UAV point cloud and photogrammetry algorithms is that UAV camera sensors are incapable of viewing beneath the canopy, which leads to sparse points and low density information of bare soil [27], whereas a single laser pulse can penetrate into an object, reach the ground, and return with multiple pulses [28]. However, because SfM and MVS are low-cost, easy to access, and easy to use, they can be efficient tools for processing UAV imagery and creating LiDAR-like point clouds [29].

Several factors affect the accuracy of point cloud datasets and consequently the digital surface model (DSM) and crop surface model (CSM) generated from them, including flight height [30], terrain morphology [31], number of ground control points (GCP) [30,32], weather conditions [33], camera type [34], UAV types (fixed-wing versus multi-rotor) [35], photogrammetry software, and algorithms [36]. For instance, Martínez-Carricondo [37] analyzed the impact of the number and distribution of GCPs on the performance of DSMs produced from UAV photogrammetry. They found that the accuracy improved and the best performance was achieved when GCPs were placed both around the edge of and inside the study area. Although performance evaluation of UAV point cloud datasets requires a comparison with LiDAR data, recently, Aboutalebi et al. [38] developed an algorithm to validate point cloud geometrical information for shaded regions detected from UAV multi-spectral imagery.

The 3D point cloud is a useful source of information about the size, position, and orientation of an object that can be combined with UAV multi-spectral or hyper-spectral imagery to explore relationships between an object’s 3D geometry information and its spectral information. Several classification methods, such as supervised and unsupervised machine learning algorithms, have been developed to generate a classified map of aerial imagery based on the similarities in spectral signatures [39]. While these algorithms fail to distinguish objects having similar spectral signatures (e.g., differentiating between water and shadows [40] in optical bands), point cloud would be a useful and an additional source to combine with multi-spectral imagery in order to improve the accuracy of classification methods. In addition to the capability of point clouds in segmentation and classification problems, point clouds are considered a crucial source of information for phenotyping.

UAV point cloud has been used to measure canopy height [41], tree height and crown diameter [42–44], to detect individual trees [45] and development of annual crops such as rice [46] and barley [47]. In addition, several studies show that bio-geophysical properties such as LAI and canopy reflectance parameters such as NDVI are correlated with above-ground biomass [48,49] and ground cover percentage [50] defined as the area of soil surface masked by plants from nadir view angle [51]. Matese et al. [52] generated a vineyard canopy height model (CHM) using an SfM point cloud and compared it with an NDVI map. They found that, although CHM from SfM underestimated canopy height (about 0.5m) due to camera resolution, it is highly correlated to NDVI maps, which means that high NDVI regions correspond to high canopy height areas. Ultimately, they estimated average volume per vine by multiplying height, width, and length of the vine canopy. Mathews and Jensen [53] explored the relationship between vineyard canopy LAI and several metrics from a UAV point cloud using a step-wise regression model. These metrics include number of points within each vine’s zone and height-based metrics (e.g., mean height of canopy). They reported a moderate positive correlation (0.57 in terms of *R*^2^) between modeled LAI and in situ measured LAI. Weisis and Baret [54] proposed a method to estimate row height, width, spacing, and vineyard cover fraction using a UAV point cloud generated from red, green, and blue (RGB) images acquired over a vineyard.

Although UAV point cloud datasets and the SfM algorithm have been widely used in characterizing vegetation structure, the full potential of the photogrammetric data has not been utilized. Most of the cited studies converted dense point cloud information into Digital Elevation Model (DEM), Digital Terrain Model (DTM), DSM, or CSM (raster versions of point cloud datasets) because working with pure LiDAR-like datasets is challenging, and algorithms and hardware that can handle such massive datasets are limited. In addition, the potential of 3D plant information to improve remote sensing-based ET models has not been explored. To the authors’ knowledge, the published studies mostly focused on assessing regression models to estimate biomass parameters such as LAI, which is a key parameter in ET models, using DSMs, CSMs, or CHMs.

In this study, we propose a methodology to incorporate the 3D information extracted from a UAV point cloud into the TSEB model. In particular, a new algorithm called Vegetation Spectral-Structural Information eXtraction algorithm (VSSIXA) is developed to extract canopy height, volume, surface area, and projected surface area (fractional cover) from the point cloud dataset without converting it to a raster file. Next, the possible relationship between in situ LAI measurements, radiometric temperature (*T*_*r*_), spectral information, and 3D derived structure parameters is explored. The sensitivity of the TSEB model to fixed values of the structural information over a vineyard block versus the spatial structural information is presented. The algorithm is evaluated from imagery and point cloud data collected by Utah State University AggieAir UAVs over a commercial vineyard located in California as part of the ARS-USDA GRAPEX Project (Grape Remote sensing Atmospheric Profile and Evapotranspiration eXperiment). Finally, the TSEB model is executed under different scenarios of LAI and other canopy biomass parameters and TSEB output are compared with flux tower measurements.

## 2. Materials and Methods

### 2.1. Site Description

**Figure 1.**
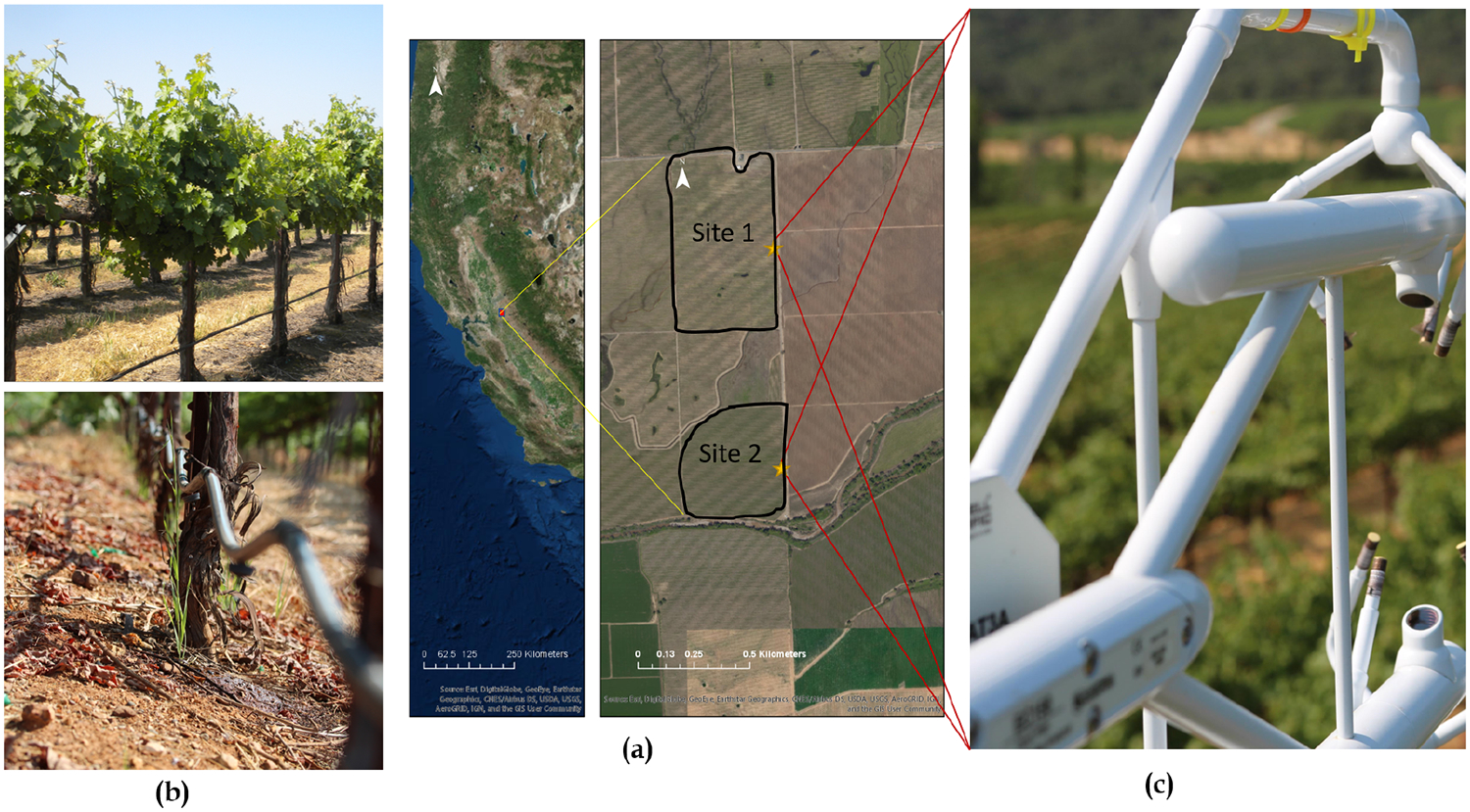
World Imagery of the study area from Environmental Systems Research Institute (ESRI) along with the locations of the flux towers (**a**), drip irrigation system (**b**), and eddy covariance instrument (**c**) installed in the area of study.

This study was conducted as a part of GRAPEX, an ongoing project started in 2013 that seeks to improve water-use efficiency through modeling of evapotranspiration and plant stress over vineyards. The vineyard test site selected is located near the town of Lodi in California’s Central Valley (38.29N, 121.12W, 38.4 m elev). This vineyard ranch called Sierra Loma (formally listed as the Borden ranch [55] consisted of two vineyard blocks, a northern and southern block, containing a flux tower in each block (Figure 1a). An overview of all continuous and episodic measurements are described in detail in [55]. The northern and southern vineyard blocks (referred to as Site 1 and Site 2 hereafter, respectively) were planted with the Pinot Noir variety in 2009 and 2011, respectively. The age differences resulted in lower vegetation density, biomass and leaf area at Site 2 compared to Site 1.

Both sites share similar trellis structure and vine management. Vines are grown on identical quadrilateral cordon fixed trellis systems with installed drip irrigation in which irrigation lines run along the base of the trellis at 30 cm above ground level (agl) with two emitters (4 L/h) between each vine. The training system employs “U” shaped trellises, and canes are trained upwards. The vine trellises are 3.35 m (11 ft) apart, and the height to first and second cordons is about 1.45 and 1.9 m, respectively [55]. Vine heights vary between 2 and 2.5 m, with space between vines of 1.5 m and an East–West row orientation. The elevated canopy included significant open space between the bottom of the canopy crown and the soil surface. This open space (~0.7 m in height during peak growing season) is occupied by the narrow trellis posts and drip irrigation line (Figure 1b).

In order to regulate soil moisture early in the growing season following the winter season, an inter-row grass cover crop is planted in both vineyards and is mowed in either late April or early May. Two flux towers were installed in 2013, one at Site 1 and another at Site 2. The towers are installed approximately half-way North–South along the Eastern edge of each site as the predominant wind direction is from the West during sunlight hours in the growing season (Figure 1c).

### 2.2. AggieAir Remote Sensing Platform

**Figure 2.**
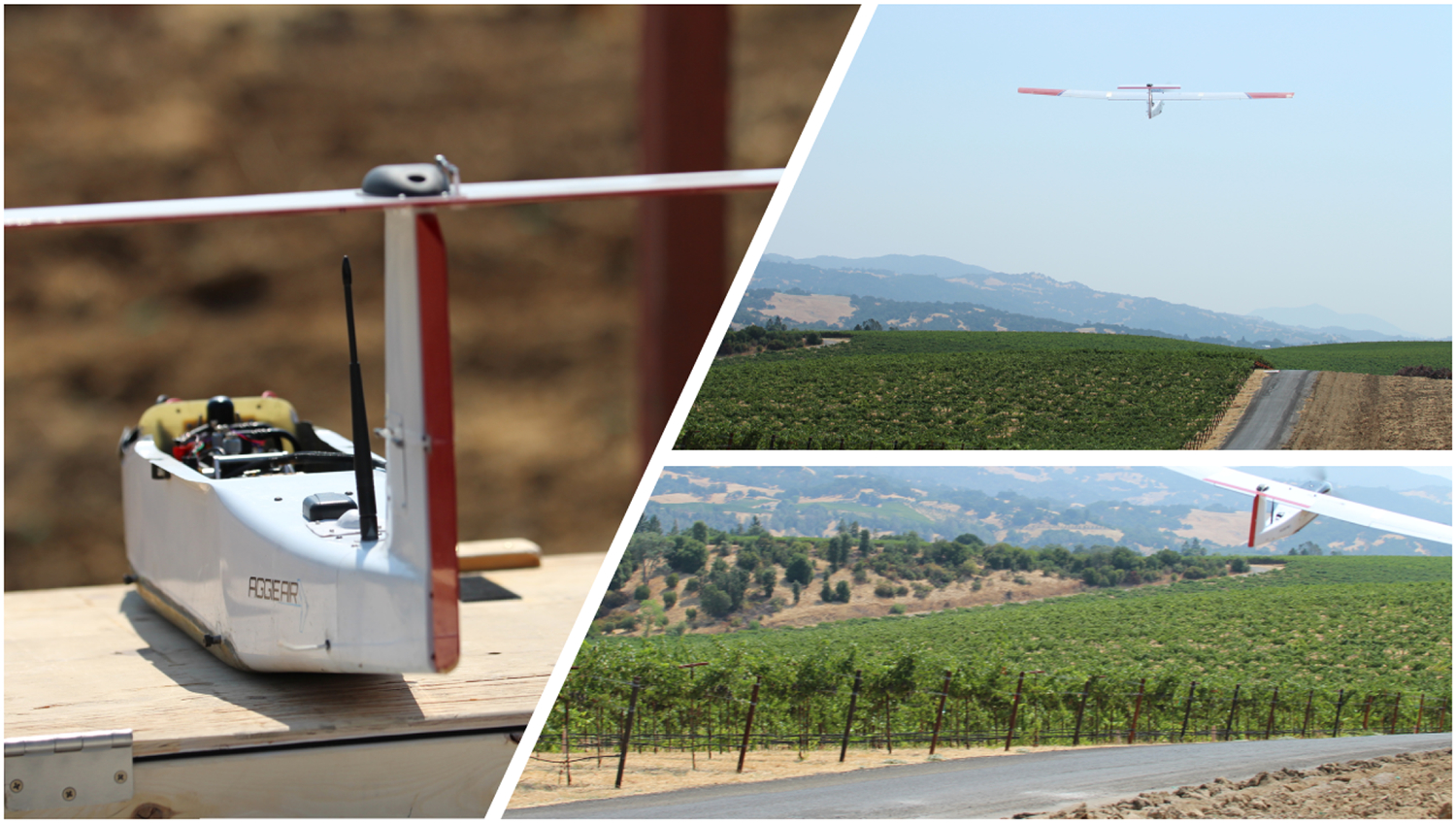
AggieAir airframe layout flying and capturing imagery over the study area.

AggieAir is a battery powered unmanned aerial vehicle (UAV) designed by Utah State University (USU) to carry multi-spectral sensor payloads and to acquire high-resolution aerial imagery at both optical and thermal spectra. This UAV platform consists of two cameras, a computer, a GPS module, an inertial measurement unit (IMU), a radio controller, and flight control, and it can be flown autonomously or manually [56]. The UAV can fly over the area of interest using a pre-programmed flight plan (in an autonomous mode) for an hour at a speed of 30 miles per hour [57], with the capability to provide very high-resolution imagery (less than 20 cm) at 1000 m agl and record the position and orientation of the aircraft when each image is taken. Figure 2 shows a layout of the AggieAir air-frame.

### 2.3. AggieAir UAV High-Resolution Imagery

**Figure 3.**
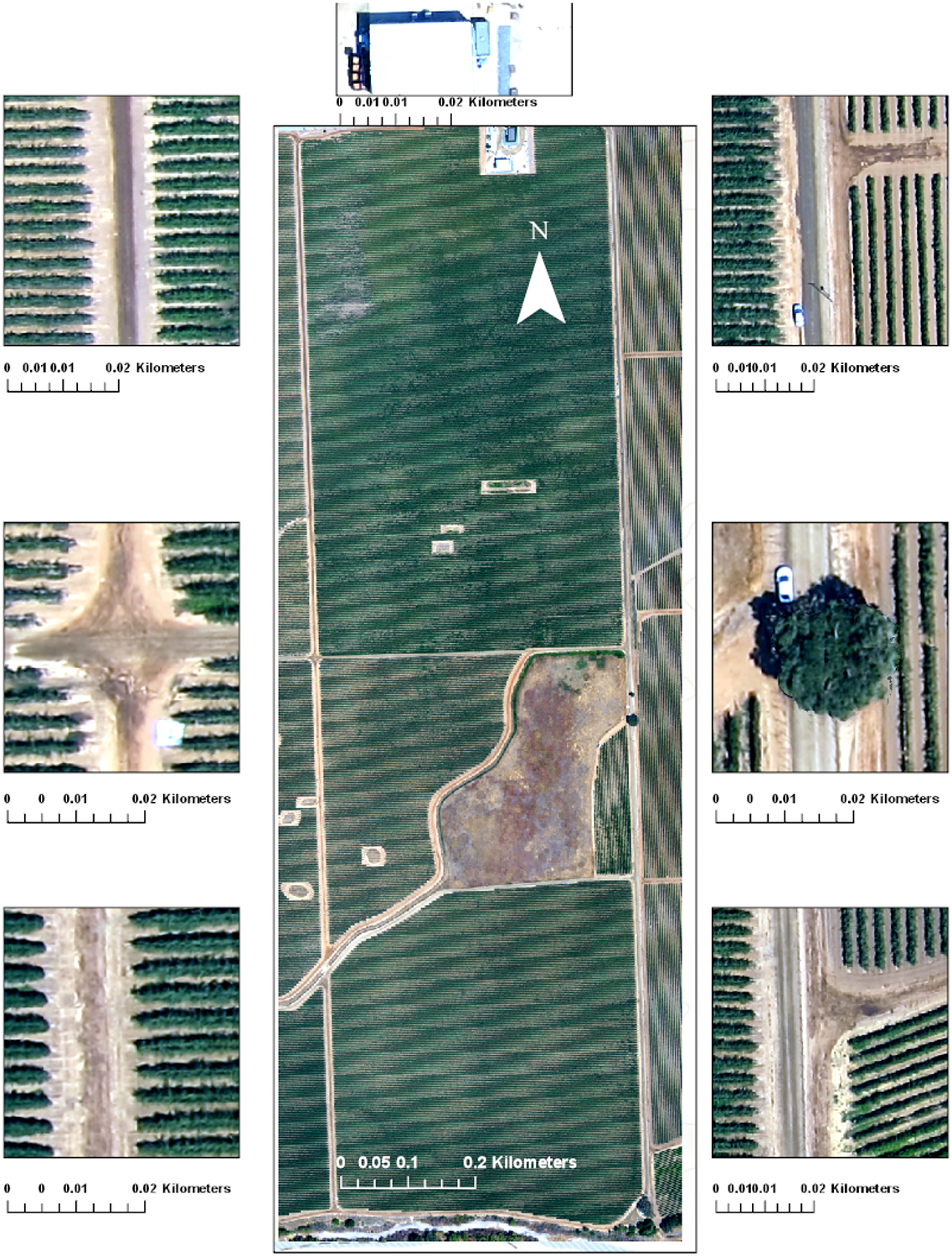
Example of high-resolution imagery captured by AggieAir over the study area in August 2014.

The high-resolution images for this study were collected by an AggieAir UAV over the GRAPEX Pinot Noir vineyard. The UAV was supplied and operated by the AggieAir UAV Research Group at the Utah Water Research Laboratory at USU [58]. Four sets of high-resolution imagery (20 cm or finer) were captured over the vineyard in 2014, 2015, and 2016. These UAV flights were synchronized with Landsat satellite overpass dates and times. A sample of the imagery captured by the UAV over the study area is shown in Figure 3, and information describing the images is summarized in Table 1.

Figure 3 shows the study area with details of sections as captured by UAV. Cameras and optical filter information, fieldwork dates, vineyard phenological stages, and imagery resolution are summarized in Tables 1 and 2.

As described in Tables 1 and 2, the imagery covers all major phenological vineyard stages. The cameras used in the current study ranged from consumer-grade Canon S95 cameras to industrial type Lumenera monochrome cameras fitted with narrowband filters equivalent to Landsat 8 specifications. The thermal resolution for all four flights was 60 cm, and the visible and near-infrared (VNIR) were 10 cm, except for the August flight.

### 2.4. AggieAir UAV Image Processing

A three-step image processing phase followed imagery acquisition. This process included (1) radiometric calibration, (2) image mosaicking and orthorectification, and (3) Landsat harmonization. In the first step, the digital images were converted into a measure of reflectance by estimating the ratio of reference images from pre- and post-flight Labsphere [59] Lambertian panel readings. This conversion method was adapted from Neale and Crowther [60]; Miura and Huete [61]; and Crowther [62] and is based solely on the reference panel readings, which do not require solar zenith angle calculations. This procedure additionally corrected camera vignetting effects that were confounded in the Lambertian panel readings. In the second step, all images were combined into one large mosaic and rectified into a local coordinate system (WGS84 UTM 10N) using Agisoft Photoscan software [63] and survey-grade GPS ground measurements. The software produced hundreds of tie-points between overlapping images by using photogrammetric principles in conjunction with image GPS log file data and UAV orientation information from the on-board IMU to refine the estimate of the position and orientation of individual images. The output of this step is an orthorectified reflectance mosaic [56]. Since different optical sensors with different spectral responses are used to capture high-resolution imagery (Table 1) and the spectral information of vegetation will be used to model LAI, a bias correction method is necessary to remove the disagreement of remotely sensed information regardless of pixel resolution and sensor. Thus, in the third step, the UAV optical high-resolution imagery was upscaled to Landsat resolution using the Landsat point spread function. If biased, it was corrected with a linear transformation [64]. For thermal imagery processing, only step 2 was applied. The resulting thermal mosaic consisted of brightness temperature in degrees Celsius. Moreover, a vicarious calibration for atmospheric correction of microbolometer temperature sensors proposed by Torres-Rua [65] was applied to the thermal images.

### 2.5. Field Measurements, Multi-Spectral Imagery, Point Cloud, and LiDAR Datasets

**Figure 4.**
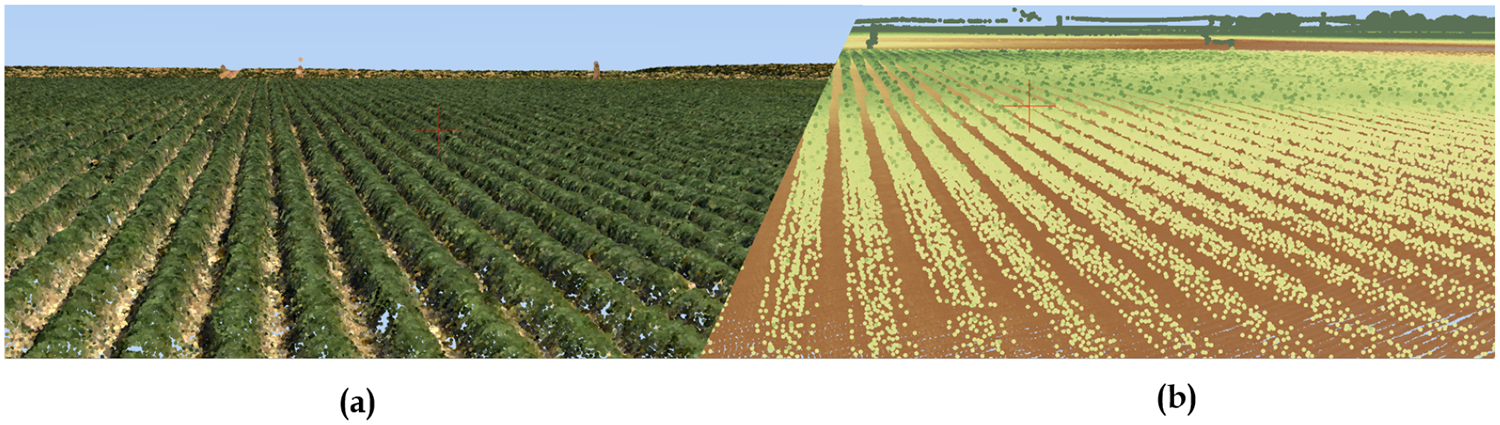
Example of a point cloud dataset produced by AgiSoft using AggieAir imagery and SfM method (**a**) versus LiDAR dataset collected by NASA G-LiHT (**b**) for the area of study.

Photogrammetric point clouds were produced from the multispectral images (Figure 4a) with a density of ~40 (points/m^2^) for the 15-cm resolution (2014 imagery) and ~100 (points/m^2^) for the 10-cm resolution (2015 and 2016 imagery), after which a DSM was generated at the same spatial resolution as the original imagery (i.e., 15 cm for 2014 and 10 cm for 2015 and 2016). In addition to UAV point cloud products that describe the surveyed surface, a LiDAR derived bare soil elevation (DTM) product for the same location, collected by the NASA G-LiHT (Goddard’s LiDAR, Hyperspectral & Thermal Imager) project in 2013, was used [66] (Figure 4b).

**Figure 5.**
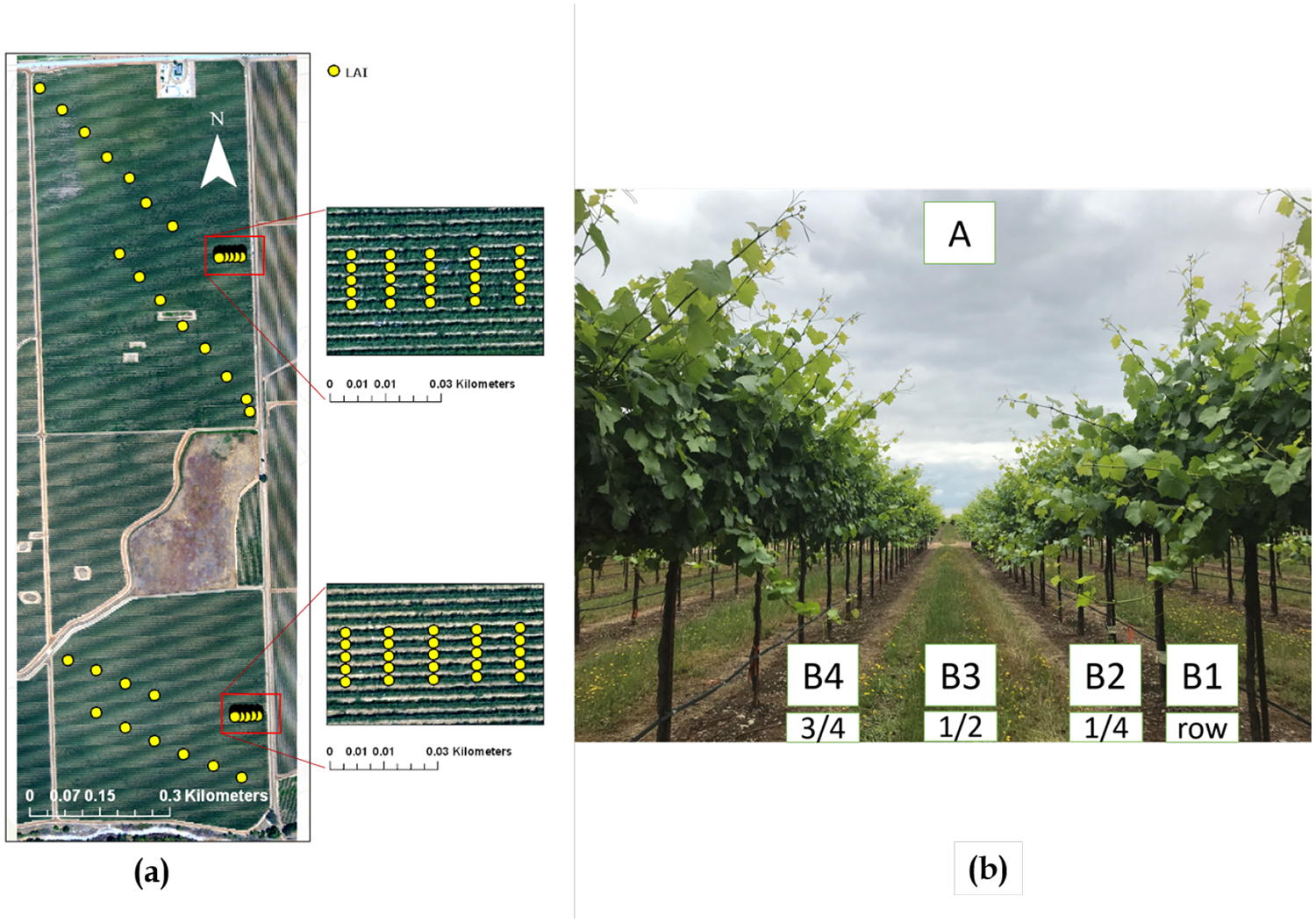
(**a**) leaf area sampling locations, (**b**) measuring LAI according to GRAPEX protocol [14].

In addition, ~80 LAI measurements for each flight were acquired using the Plant Canopy Analyzer (PCA, LAI2200C, LI-COR, Lincoln, NE, USA) as the indirect in situ LAI measurements (Figure 5). These LAI measurements were validated with direct LAI (i.e., destructive sampling) measurements [14].

**Figure 6.**
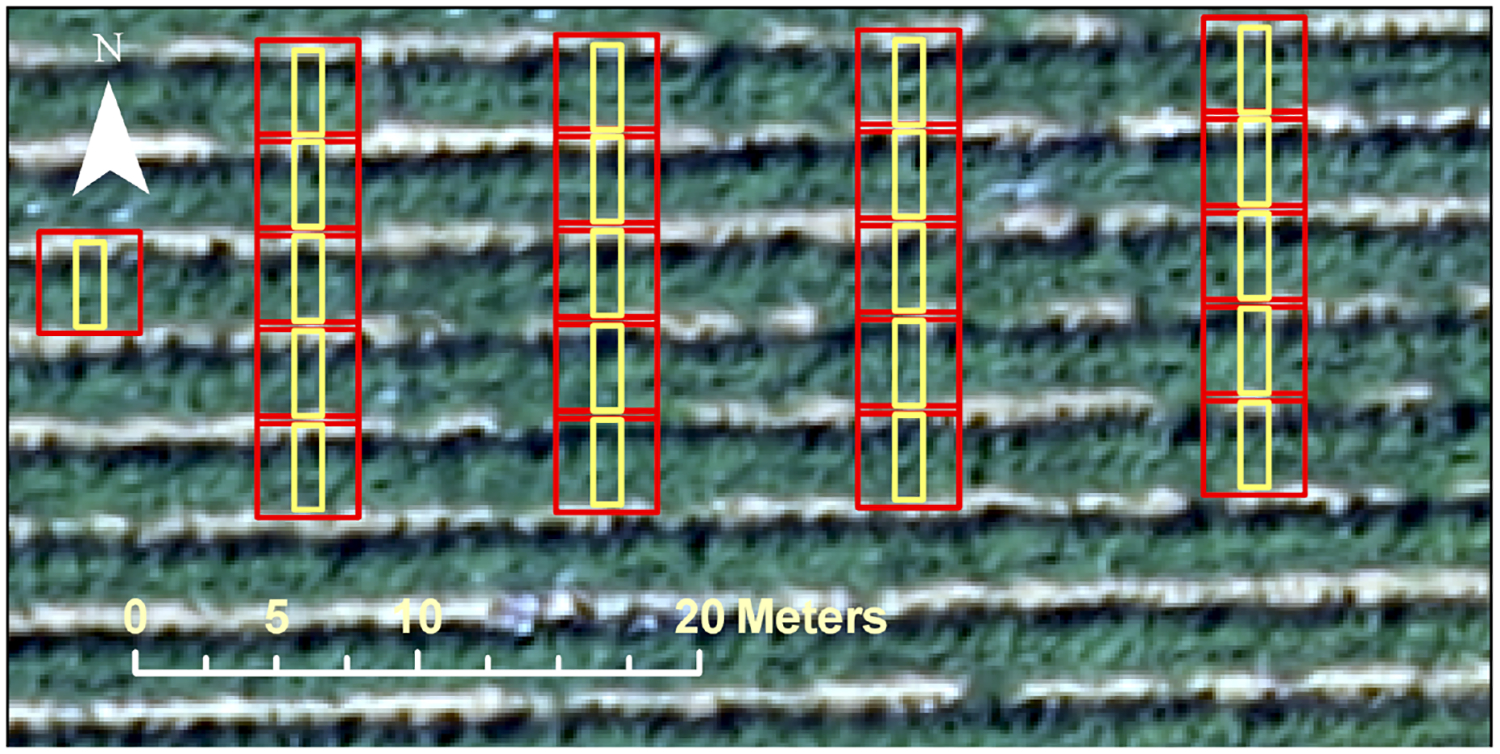
Square and rectangle buffers around LAI measurements.

The location of each measurement is recorded with a precise Real-time kinematic (RTK) GPS (Figure 5). To evaluate the relationship between vine spectral-structural information and in situ LAI measurements, first the footprint of the LICOR-2200C must be defined. According to White et al. [14], it was assumed that the LICOR-2200C was measuring LAI in a rectangle 1 m wide and 3 m long. However, the smallest valid resolution in applying the TSEB model for the study area was determined to be 3.6-m grid [67], which means that all required inputs for the TSEB model must be set to 3.6-m grids. Due to inconsistency between the LICOR-2200C footprint and the TSEB model resolution and its unknown impact on the LAI map, vine spectral-structural information is extracted for both rectangular and square buffers around LAI measurements (Figure 6).

Eddy covariance and micrometeorological data, surface fluxes, and meteorological conditions are being collected year round at each of the vineyard sites for starting in 2013. The raw high-frequency data have been fully processed and evaluated for quality control and are stored as hourly block-averaged data. Wind speed and wind direction are measured via sonic anemometer (CSAT3, Campbell Scientific) mounted 5 m agl facing due west (270°). Air temperature is measured via a humidity/temperature sensor (HMP45C, Vasaila) mounted at 5 m agl. Water vapor density is measured via a humidity/temperature sensor (HMP45C, Vasaila) mounted at 5 m agl. Atmospheric pressure is measured by a pressure sensor (EC150, Campbell Scientific) mounted 5 m agl facing due west (270°). Incident long-wave radiation and net radiation are measured via a 4-component net radiometer (CNR-1, Kipp & Zonen,) mounted 5 m, agl facing southwest (225°). Sensible and latent heat flux are derived from CSAT and EC150 data. Soil heat flux is the mean of the five measurements collected along a transect across the inter-row.

For the post-processing of the turbulent fluxes, the high-frequency data was screened to identify and remove flagged values (CSAT or infrared gas analyzer (IRGA) diagnostic), physically unrealistic values, and statistical outliers (data spikes). The sonic temperature was converted to air temperature following Schontanus [68] and Lui [69]. The measurements of the wind velocity components were rotated into the mean streamwise flow following the 2D coordinate rotation method described by Tanner and Thurtell [70]. The wind velocity and the scalar quantities were adjusted in time to account for sensor displacement and optimize the covariance. The frequency response correction of Massman [71] was applied. The turbulent fluxes were calculated. The initial estimates of the latent heat flux and the carbon dioxide flux were then corrected for density effects following the Webb et al. method [72]. The initial estimates of the sensible heat flux were corrected for buoyancy effects [73]. The soil heat flux was corrected for heat storage in the overlying soil layer [74]. The data were quality controlled via visual inspection to remove physically unrealistic values due to rainfall, dew, and similar events. Output of fluxes and ancillary micrometerorlogical data are stored as hourly block-averaged data.

Traditionally, any imbalance of net radiation (Rn) - soil heat flux (G) versus sensible heat flux (H) + latent heat flux (LE) is considered a lack of energy balance closure. It is often assumed that H and LE have been underestimated by the eddy covariance method, and the level of underestimation is often used to indicate the reliability of the eddy covariance estimates of H + LE [75]. The value of the ratio of (Rn-G)/(H+LE) should ideally be equal to 1, but, generally, values over 0.80 are considered reliable [75,76]). In this study, for any imbalance between Rn-G and H+LE, closure was forced by assuming that the Bowen ratio *H*/*LE* is correct because both are probably underestimated. Moreover, recent studies indicate that flow distortion for non-orthogonal sonics underestimate vertical wind and hence the turbulent fluxes [77–80]. Therefore, energy is added to H and LE (*H*_*BR*_ and *LE*_*BR*_) according to the Bowen ratio (BR) to reach a closure value of 1.0; this is typically called forcing energy balance closure [75]. Therefore, H and LE from eddy covariance are modified by Equations (1) and (2):

(1) $$H_{BR}=\frac{H}{H+LE}\times (Rn-G-H-LE)+H,$$

(2) $$LE_{BR}=\frac{LE}{H+LE}\times (Rn-G-H-LE)+LE.$$

### 2.6. Vegetation Structural-Spectral Information Extraction Algorithm (VSSIXA)

**Figure 7.**
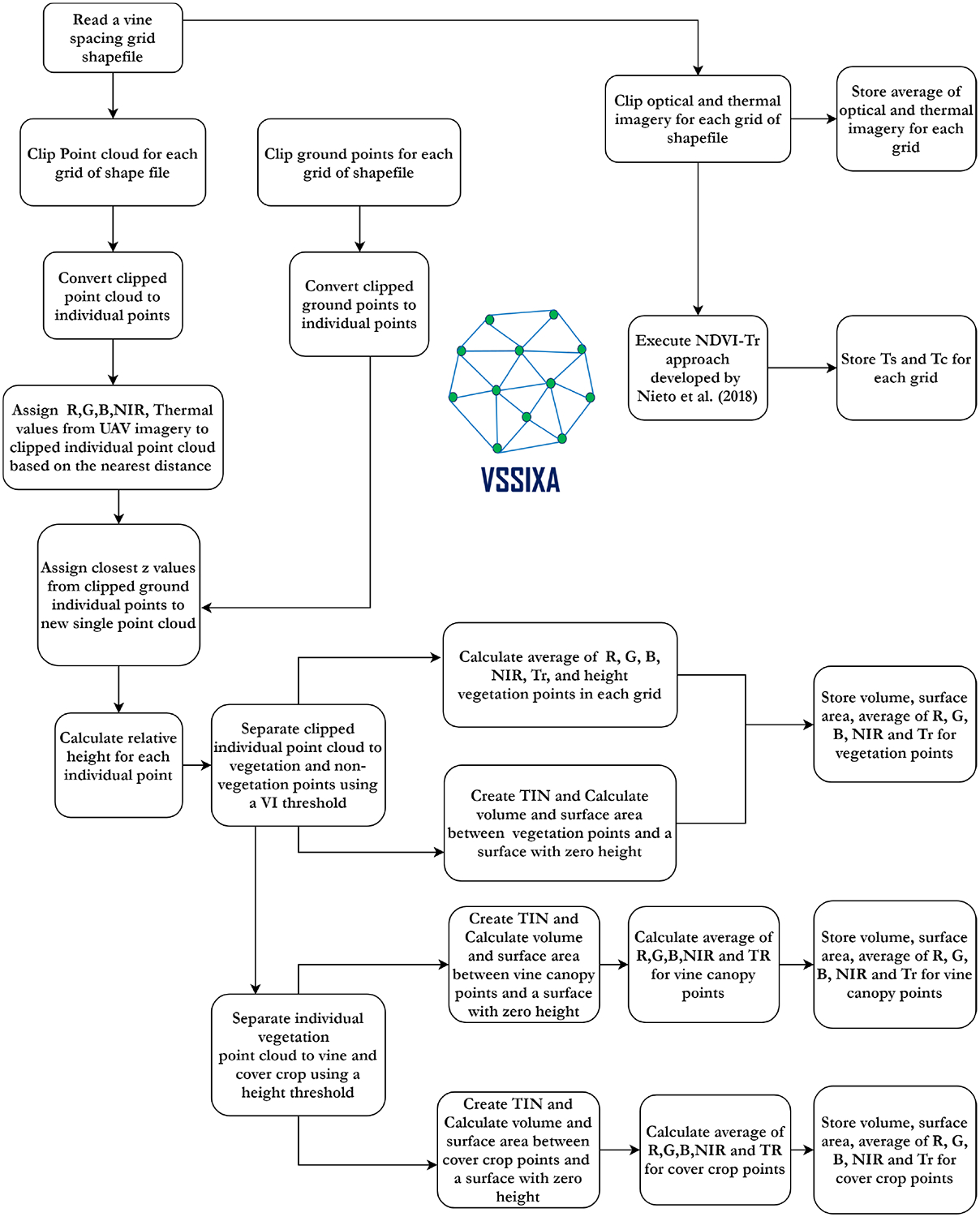
A workflow of proposed VSSIXA algorithm.

To analyze and extract 3D information from the point cloud dataset and spectral information from the high-resolution imagery, a new algorithm called Vegetation Structural-Spectral Information eXtraction Algorithm (VSSIXA), using Python and ArcGIS Pro libraries, was developed. The code of this algorithm is available at [81]. Figure 7 shows components of VSSIXA in a flowchart diagram.

As shown in Figure 7, the VSSIXA algorithm requires a point cloud dataset as the primary input and a shapefile, optical and thermal imagery, and a ground point as the secondary inputs. In the first step, a vine spacing grid shapefile is read and point cloud, ground points, and UAV imagery are clipped for each grid of the shapefile. In this step, the average of the UAV imagery for each band and for each grid, and consequently the partitioning of Tr into soil temperature (Ts) and canopy temperature (Tc) are executed and stored. In this step, Ts and Tc estimations are by-products of VSSIXA. Next, clipped ground points and point cloud datasets are converted to individual point datasets, Red (R), Green (G), Blue (B), near-infrared (NIR), and Tr bands from UAV imagery along with *z*-values from ground points are assigned to each single point cloud based on nearest distance, and relative height (Point cloud z-Ground z) is calculated. Therefore, the Attribute Table of each point constitutes point cloud height, ground height, relative height, RGB, NIR, and thermal information. Next, the individual points are separated into vegetation and non-vegetation points using a VI threshold (e.g., NDVI > 0.6), and volume, surface area, height, and the average of Tr and optical bands for vegetation points using a triangulated irregular network (TIN) are calculated and appended into the Attribute Table. In the last stage, vegetation points are separated into vine canopy and cover crop points based on a relative height threshold (0.5 m in this study) and derived structural and spectral information for vine and cover crop points is separately recalculated. Because structural and spectral information for each point has been extracted and geographical information for those single points has been accessed, a profile of information, such as average height, vine temperature, and VIs, can be extracted. VSSIXA is able to extract and store these profiles in a comma-separated values (CSV) format.

**Figure 8.**
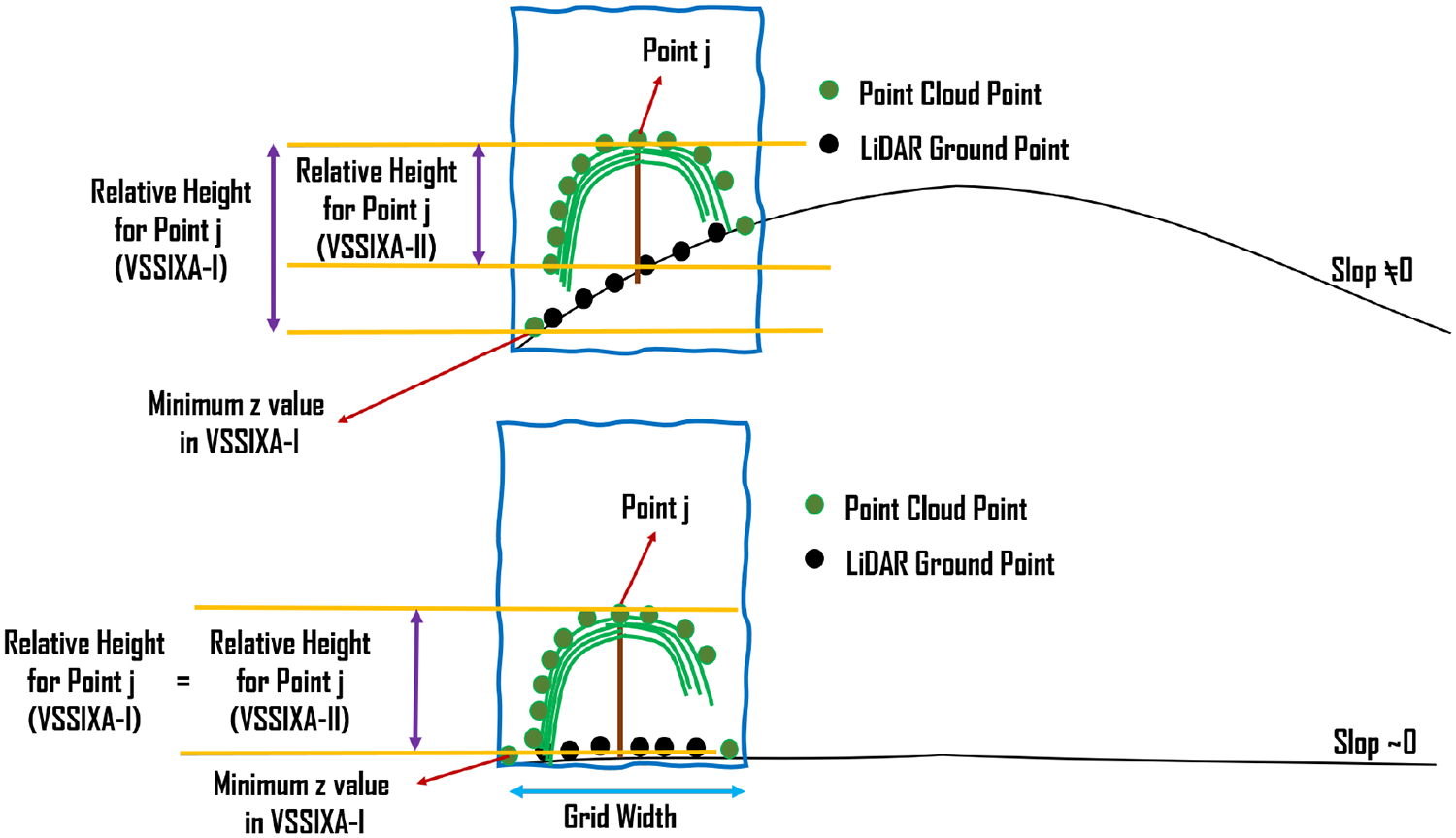
Differences between VSSIXA-I and VSSIXA-II determination of ground elevation and canopy height.

VSSIXA is coded in two different versions, VSSIXA-I and VSSIXA-II. VSSIXA-I requires only a point cloud dataset, while VSSIXA-II requires both point cloud data and LiDAR ground points. In VSSIXA-I, after appending multi-spectral information to each point in each grid, the point cloud is classified into the ground and non-ground classes based on an NDVI threshold. The relative height is calculated based on Point Cloud z and the minimum value of ground point heights. Therefore, the structural information is calculated between TIN created from non-ground points and a surface with height zero. If there are no multi-spectral data to separate ground points from non-ground points or if a grid has no ground points (e.g., fully covered by vegetation), VSSIXA-I considers the minimum *z*-value from all points to calculate relative height. In contrast, the classified ground points exist for VSSIXA-II, due to LiDAR penetration into vegetation and detection of ground. Therefore, *z*-values from LiDAR ground points are affixed to the point cloud from a spatial perspective (e.g., closest distance) to calculate relative height and then, similar to VSSIXA-I, the structural information is calculated. Since VSSIXA-I assigns one value (minimum z value of ground points) to non-ground points in each grid, it assumes that the slope of the ground surface in each grid is close to zero. Thus, VSSIXA-I is appropriate for flat terrain, even though it requires only a point cloud dataset. In contrast, because VSSIXA-II assigns ground z values to each point, the impact of slope is considered, albeit it requires both point cloud and LiDAR ground point datasets (Figure 8).

**Figure 9.**
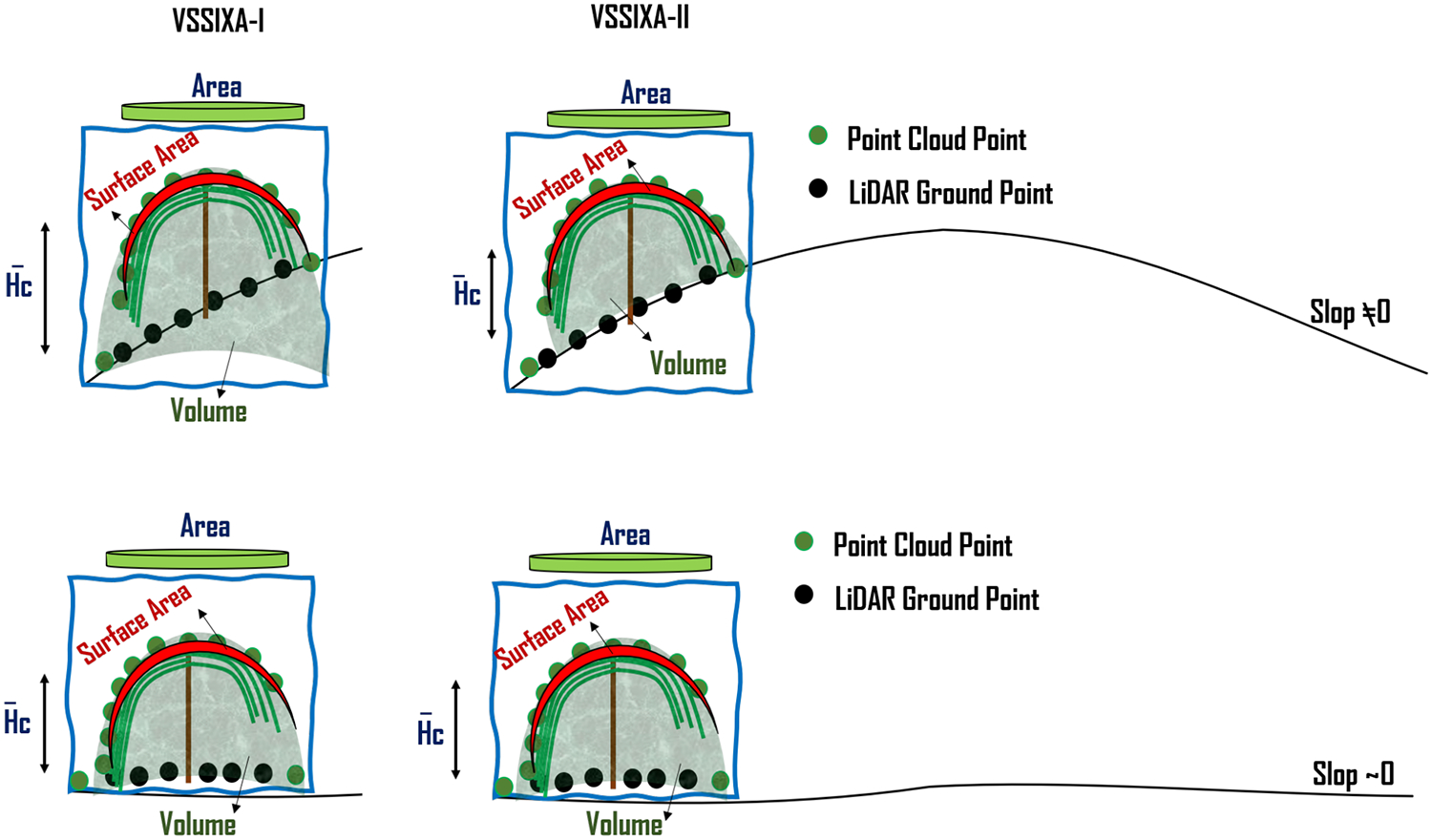
Differences between VSSIXA-I and VSSIXA-II in estimation of canopy surface area, projected surface area, volume, and average height.

The difference between VSSIXA-I and VSSIXA-II in relative height calculation may lead to differences in the estimation of canopy volume. It is expected that VSSIXA-II estimates higher values for canopy volume compared to VSSIXA-I. In contrast, there should not be a significant difference between surface area or projected surface area estimated by VSSIXA-I and VSSIXA-II (Figure 9). Thus, if all the structural parameters are used to evaluate the relationship between LAI and VSSIXA outputs, either VSSIXA-I or VSSIXA-II must be employed for the entire study area due to inconsistency between canopy volume and height estimated by VSSIXA-I and -II unless the slope of each grid can be considered as zero (similar to the current study area).

#### Genetic Programming: GP

**Figure 10.**
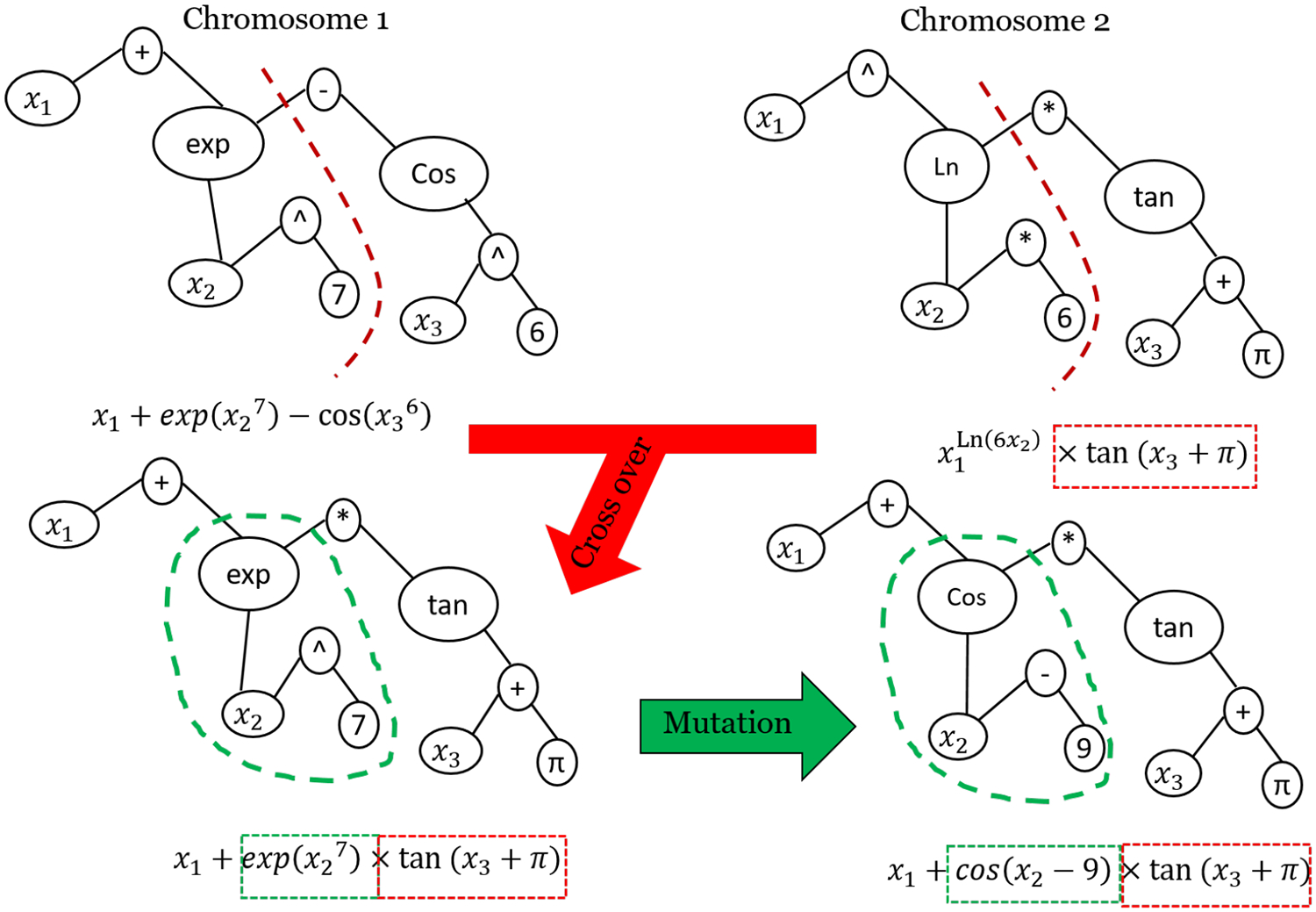
A graphical visualization of the various stages of GP to update solutions (chromosomes).

Genetic Programming (GP) is a machine learning method inspired by the genetic algorithm (GA). In contrast to a trained network with Artificial Neural Network (ANN) and Support Vector Machine (SVM), the output of GP is a trained equation that researchers can simply use and calibrate in different study areas. Similar to GA, GP uses a searching process to solve optimization problems. It starts with many possible solutions in the form of chromosomes, in which each gen could be a function (*sin*, *log*, *cos*, and *exp*), an operator (+,−, /), an input variable (*x*_1_, ⋯ , *x*_*n*_), or a number (1, 2, 3, ⋯ , n). In iteration 1, chromosomes (equations) are generated by a random initial solution. Then, chromosomes are ranked (from the best to the worst) based on an objective function (e.g., Root Mean Square Error (RMSE) calculated for each chromosome. In other words, input data $\left(\vec{X}=x_{1},\cdots ,x_{n}\right)$ are input to each chromosome (equation) to calculate outputs $\left(f_{1}(\vec{X}),\cdots ,f_{n}(\vec{X})\right)$; the outputs of each chromosome $\left(f_{1}(\vec{X}),\cdots ,f_{n}(\vec{X})\right)$ are compared with observed values (*y*_1_, ⋯ , *y*_*n*_); an objective function (e.g., RMSE) is calculated for each chromosome (equation); and these initial solutions are sorted based on objective function values. In subsequent iterations, solutions (chromosomes) must be updated. Each chromosome can be modified in each iteration of the search process using cross-over and mutation functions. Cross-over is responsible for interpolation between two chromosomes, and mutation is designed for extrapolation. In each iteration, if the stopping criteria (e.g., number of iterations *<* 1e6) is satisfied, GP will stop, and the first among the sorted chromosomes, which is a fitted linear or nonlinear equation, is reported as the best solutions. Figure 10 shows the evolving process for one chromosome after one iteration using mutation and cross-over functions.

In this study, spectral-structural information (e.g., canopy volume and surface area) estimated by VSSIXA for each in situ LAI domain (input dataset) and in situ LAI (output dataset) is used train GP. Thus, GP is employed to search possible linear and nonlinear relationships (equations) between VSSIXA outputs (e.g., canopy volume and surface area) and in situ LAI in order to create an LAI map for the TSEB model.

One of the advantages of GP is access to a formula in which inputs are related to outputs, whereas the trained networks of popular machine learning methods such as ANN and SVM do not explicitly provide a formula, only results and performances. Without access to trained networks (weights, bias, and sometimes kernel parameters), reproducing results or evaluation of the performance of the trained network for a different case study is not possible. In contrast, the trained network of GP is reported in the form of an equation (sometimes a complex equation). This feature makes GP a tool [82] with a transferable trained network, although the proposed GP models should be confirmed under different planting geometries, and local calibration may be needed.

A software called “Eureqa” [83,84] is used to execute GP, wherein 70% of the dataset records are considered for training the network, and 30% are allocated for the testing procedure. To train GP, basic (e.g., +,−,*,/), trigonometric (sin, cos), and exponential formula building-blocks are used, and maximizing R-square is considered the objective function.

### 2.7. TSEB-2T Model

**Figure 11.**
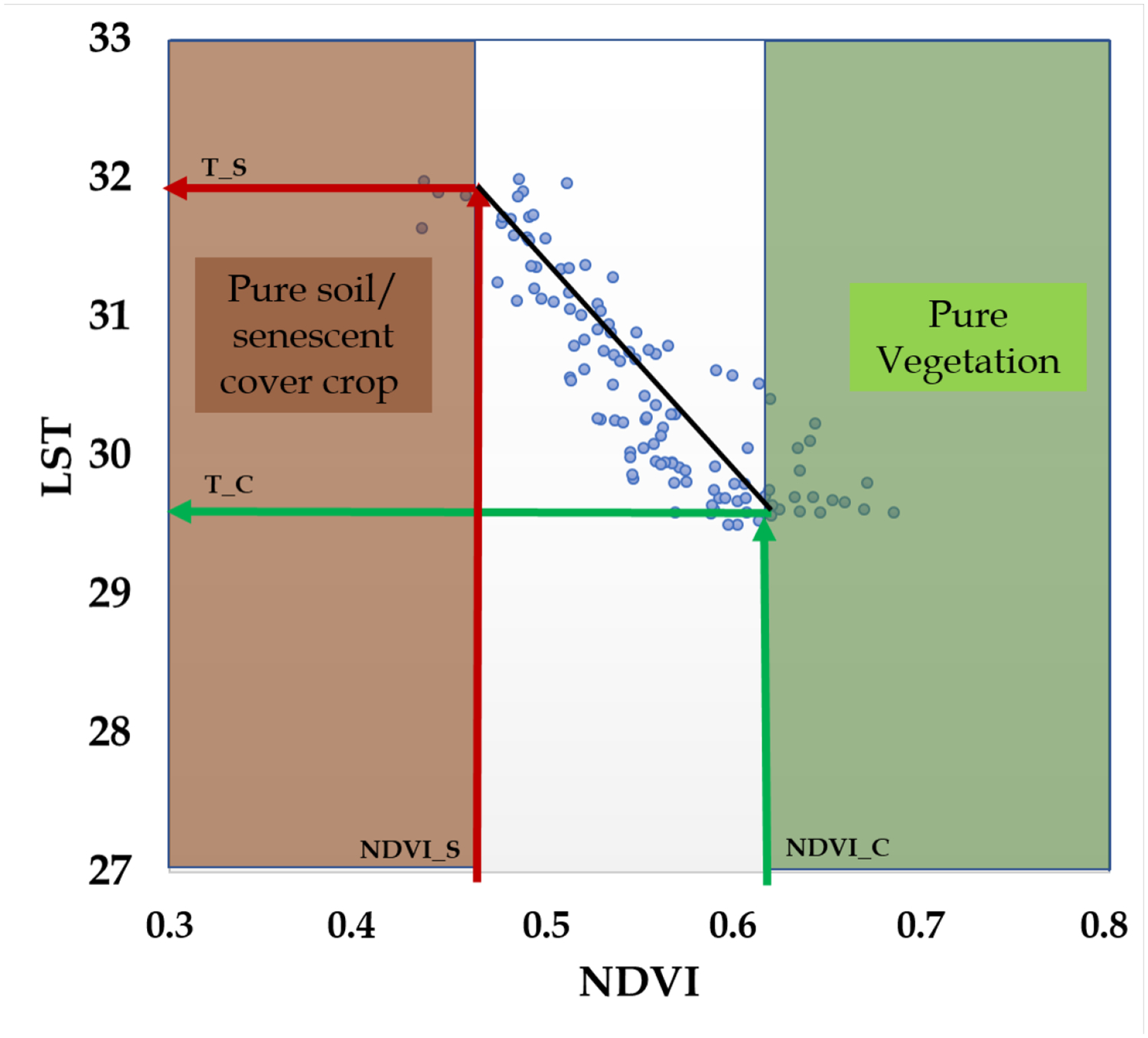
Example of a contextual NDVI-Trad scatterplot used for searching Ts and Tc within a 3.6-m grid.

TSEB-2T is a version of the TSEB model that was developed for when both Ts and Tc can be derived from nadir and off nadir Tr viewing angles [85] or by deriving pure vegetation and soil/cover crop pixels in a contextual spatial domain, namely VI-Tr space [67]. The contextual domain is a 3.6 × 3.6 m grid mapping NDVIs versus Tr (Figure 11). Next, a linear function via least squares regression is fit to the NDVI-Tr pairs. Pure vegetation and soil/cover crop pixel values are defined using histogram analysis or an LAI-NDVI empirical relationship for the entire field. These threshold values are substituted into the fitted linear equation, and two temperatures are retrieved. The lowest and highest temperatures are assigned for Tc and Ts, respectively.

In addition to Ts and Tc, TSEB requires LAI, fractional cover, soil and canopy emissivity, albedo, information of the canopy structure (leaf width, canopy height), and atmospheric forcing, air temperature (Ta), wind speed coming, solar radiation and vapor pressure. VSSIXA is able to produce LAI, fractional cover, and canopy structure information such as canopy height based on the point cloud information. Without VSSIXA, LAI is estimated based on empirical relationships between VIs and in situ LAIs, and fractional cover and canopy height are fixed values for the entire domain.

**Figure 12.**
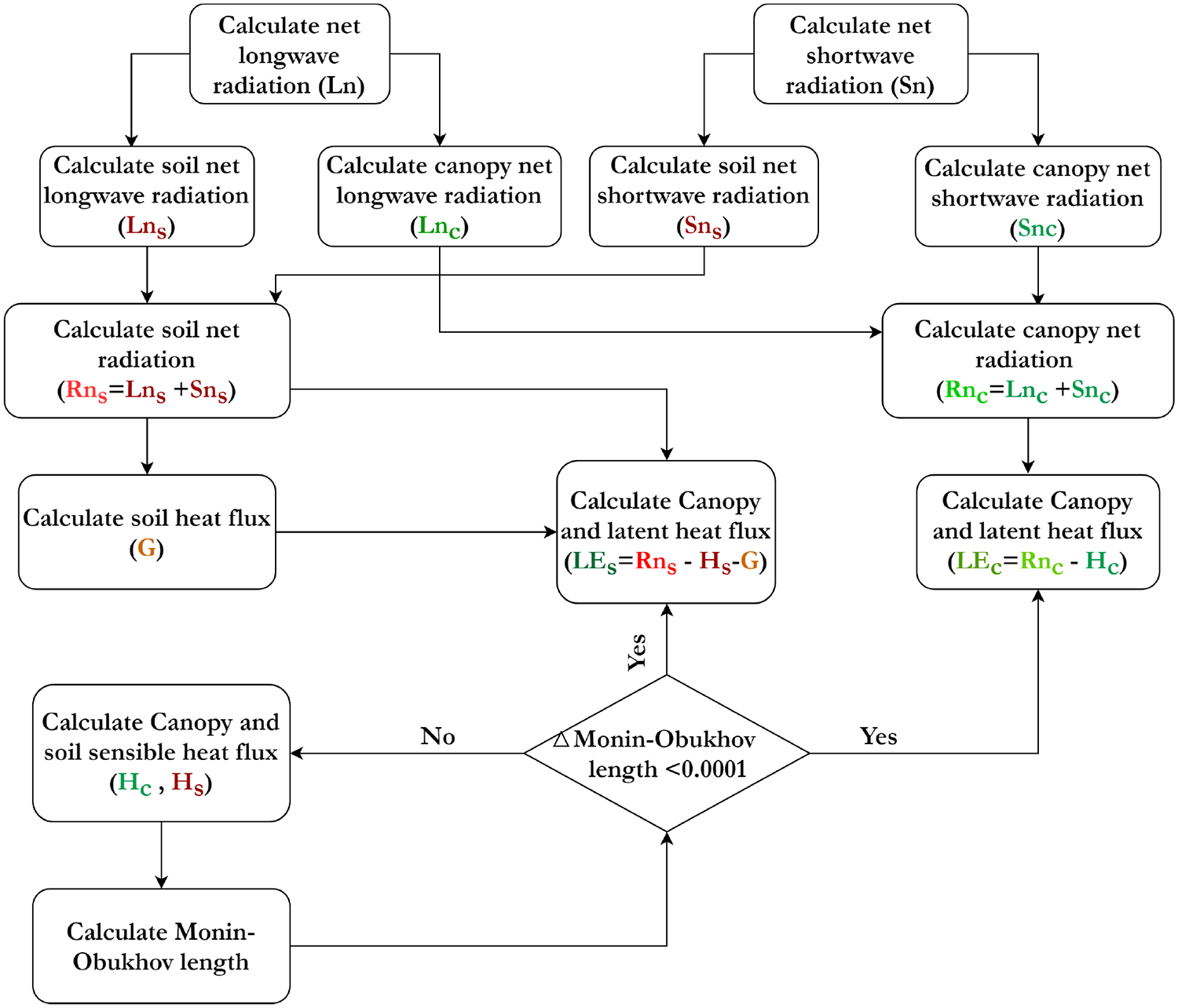
Connections between TSEB model components for the energy fluxes calculation.

In TSEB with Tc and Ts estimates (Figure 12) using the TSEB-2T version [67,85], net shortwave (*Sn*) and longwave radiation (*Ln*) are generally calculated at the first steps. Next, net longwave radiation is separated into canopy and soil net longwave radiation (*Ln*_*s*_ and *Ln*_*c*_) using a formulation developed by Kustas and Norman [86] (Equations (3) and (4)):

(3) $$Ln_{c}=\left(1-exp\left(-k_{L}\Omega LAI\right)\right)\left(L_{sky}+L_{s}-2L_{c}\right),$$

(4) $$Ln_{s}=exp\left(-k_{L}\Omega LAI\right)L_{sky}+\left(1-exp\left(-k_{L}\Omega LAI\right)\right)L_{c}-L_{s},$$

where *k*_*L*_ is the long-wave radiation extinction coefficient, Ω is the vegetation clumping factor proposed by [86], and *L*_*s*_, *L*_*c*_ and *L*_*sky*_ (*W*/(*m*^2^)) are the long-wave emissions from soil, canopy and sky, respectively.

In addition, net shortwave radiation is separated into canopy and soil net shortwave radiation (*Sn*_*s*_ and *Sn*_*c*_) based on the canopy radiative transfer model developed by Campbell and Norman [87]. Then, net radiation at the soil and canopy are calculated based on the summation of net longwave and shortwave radiation for each component (*Rn*_*s*_ and *Rn*_*c*_; Equations (5) and (6)):

(5) $$Rn_{c}=Ln_{c}+\left(1-\tau_{s}\right)\left(1-\alpha_{c}\right)S,$$

(6) $$Rn_{s}=Ln_{s}+\tau_{s}\left(1-\alpha_{s}\right)S,$$

where *τ*_*s*_ is solar transmittance through the canopy, S (*W*/(*m*^2^)) is the incoming short-wave radiation, *α*_*c*_ and *α*_*s*_ are the canopy and soil albedo, respectively.

Since soil heat flux (*G*) is assumed to be a portion of *Rn*_*s*_ (e.g., 30%), it is simply computed at this step. Next, sensible heat flux is estimated for the canopy and soil components (*H*_*s*_ and *H*_*c*_) initially assuming a neutral atmospheric stability, but it is corrected in an iterative loop until changes in the Monin–Obukhov stability length scale reach a minimum (i.e., changes between consecutive calculations of the Monin–Obukhov length is less than 0.00001). Ultimately, latent heat flux for soil and canopy (*LE*_*s*_ and *LE*_*c*_) are calculated as residuals of the soil and canopy energy balance equations, namely Equations (7) and (8), respectively:

(7) $$LE_{S}=Rn_{S}-G-H_{S},$$

(8) $$LE_{C}=Rn_{C}-H_{C}.$$

### 2.8. Data Analysis

The relationship between VSSIXA outputs and in situ LAI measurements, as well as the accuracy of the TSEB model considering different inputs against eddy covariance measurements, is evaluated using coefficient of determination (*R*^2^), mean absolute error (MAE), RMSE, and relative root mean square error (RRMSE) (Equations (9)–(12)):

(9) $$R^{2}=1-\frac{\sum_{i=1}^{n}{\left(M_{i}-E_{i}\right)}^{2}}{\sum_{i=1}^{n}{\left(M_{i}-{\bar{M}}_{i}\right)}^{2}},$$

(10) $$MAE-\frac{\sum_{i-1}^{n}\left|M_{i}-E_{i}\right|}{n},$$

(11) $$RMSE=\sqrt{\frac{\sum_{i=1}^{n}{\left(M_{i}-E_{i}\right)}^{2}}{n}},$$

(12) $$RRMSE-\frac{RMSE}{{\bar{M}}_{i}}\times 100,$$

in which *n* is the number of observations, *M*_*i*_ is measured value, *E*_*i*_ is estimated value, and ${\bar{M}}_{i}$ is the average of measured values. *R*^2^ is often used to estimate the performance of the models and shows the fraction of the estimated values that are closest to measurement data. MAE is an indicator for average model performance error and is less sensitive to outliers [88]. RMSE is designed to show the predictive capability of a model in terms of its absolute deviation [89]. RRMSE is a dimensionless version of RMSE, and model accuracy is connoted excellent when RRMSE < 10%, good if 10%< RMSE < 20%, fair if 20% < RMSE < 30% and poor if RRMSE > 30% [90].

## 3. Results

### 3.1. VSSIXA Outputs

**Figure 13.**
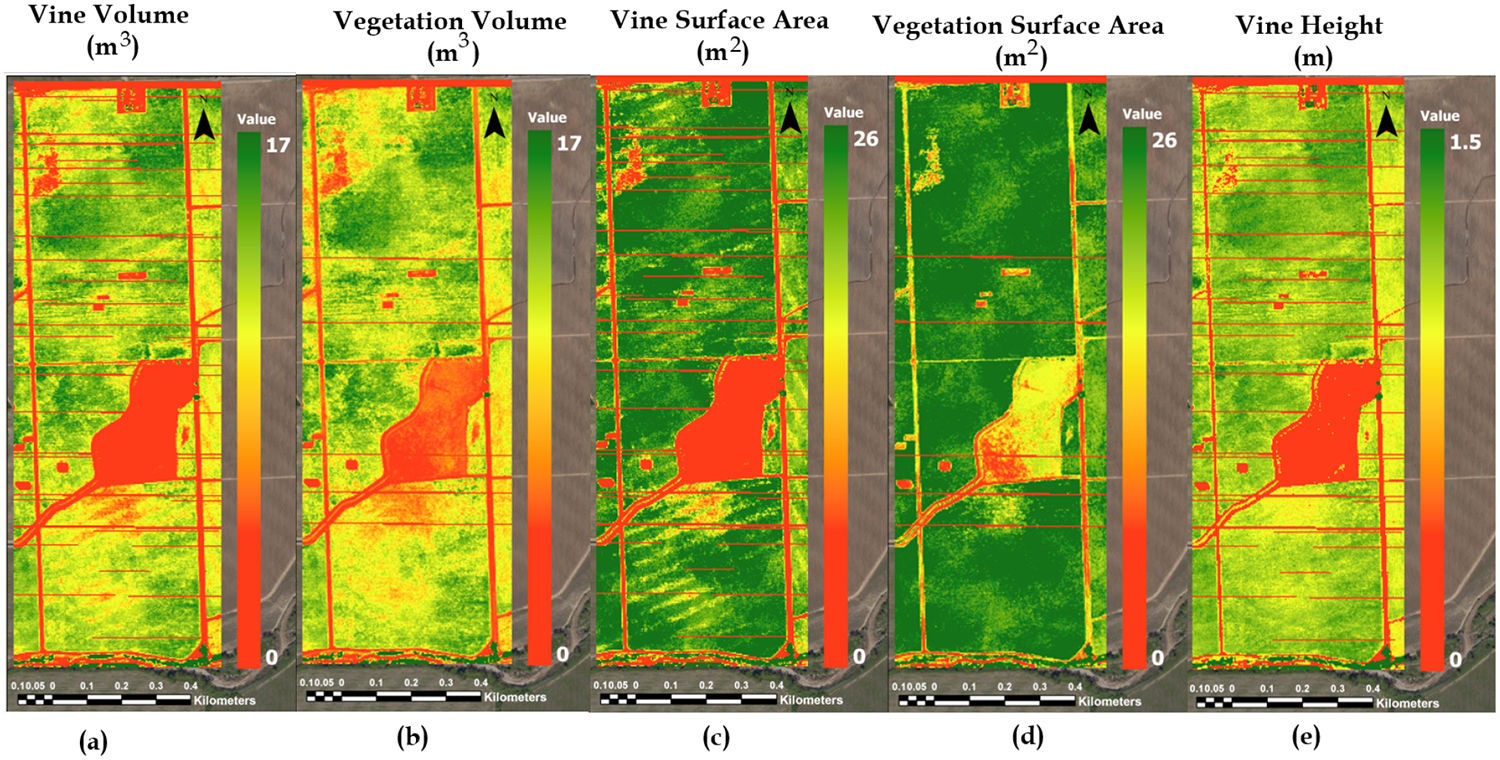
Examples of (**a**) vine volume, (**b**) vegetation volume, (**c**) vine surface area, (**d**) vegetation surface area, (**e**) vine height and cover crop height calculated for a 2015 July point cloud dataset using VSSIXA-II (horizontal lines are areas of missing data).

VSSIXA is able to provide information such as canopy height, volume, surface area, and projected surface area (PSA) directly from the point cloud data. Due to the presence of both grass cover crop and grapevine canopy in the study area, a 0.5-m threshold is considered to separate grapevine canopy from grass. After the separation, the vegetation structure information is executed for three categories: (1) vine canopy, (2) cover crop, and (3) vegetation (both vine canopy and cover crop). Examples of this information derived from a 2015 July point cloud dataset is shown in Figure 13.

Vegetation volume and vine volume (Figure 13) show similar patterns, indicating Site 1 (northern site) clearly has higher biomass compared to Site 2 (southern site). These differences in biomass amount are likely related to the difference in age, with vines at Site 1 more mature than Site 2. The grapevines planted in Site 1 have greater height and surface area versus those planted in Site 2. As expected, canopy volume, height, and surface area values in an area between the north and south blocks and roads are close to zero since these areas contain no grapevine. Although zero plant height regions are not of interest in this study, these zero height values do show the accuracy of the point cloud data since overlaying the high resolution imagery of Figure 3 has a very high correspondence with roads and the non-vineyard field separating north and south vine blocks. Low, dense, and short vegetation in the area separating the two vineyard blocks, which is visible in Figure 3, appeared in vegetation volume and vegetation surface area maps (Figure 13b,c). The horizontal lines of missing data are due to a lack of sufficient data points in the UAV point cloud acquisition and are probably a result of inadequate overlapping in the UAV imagery. This can be solved by increasing the overlap in adjacent image acquisitions.

**Figure 14.**
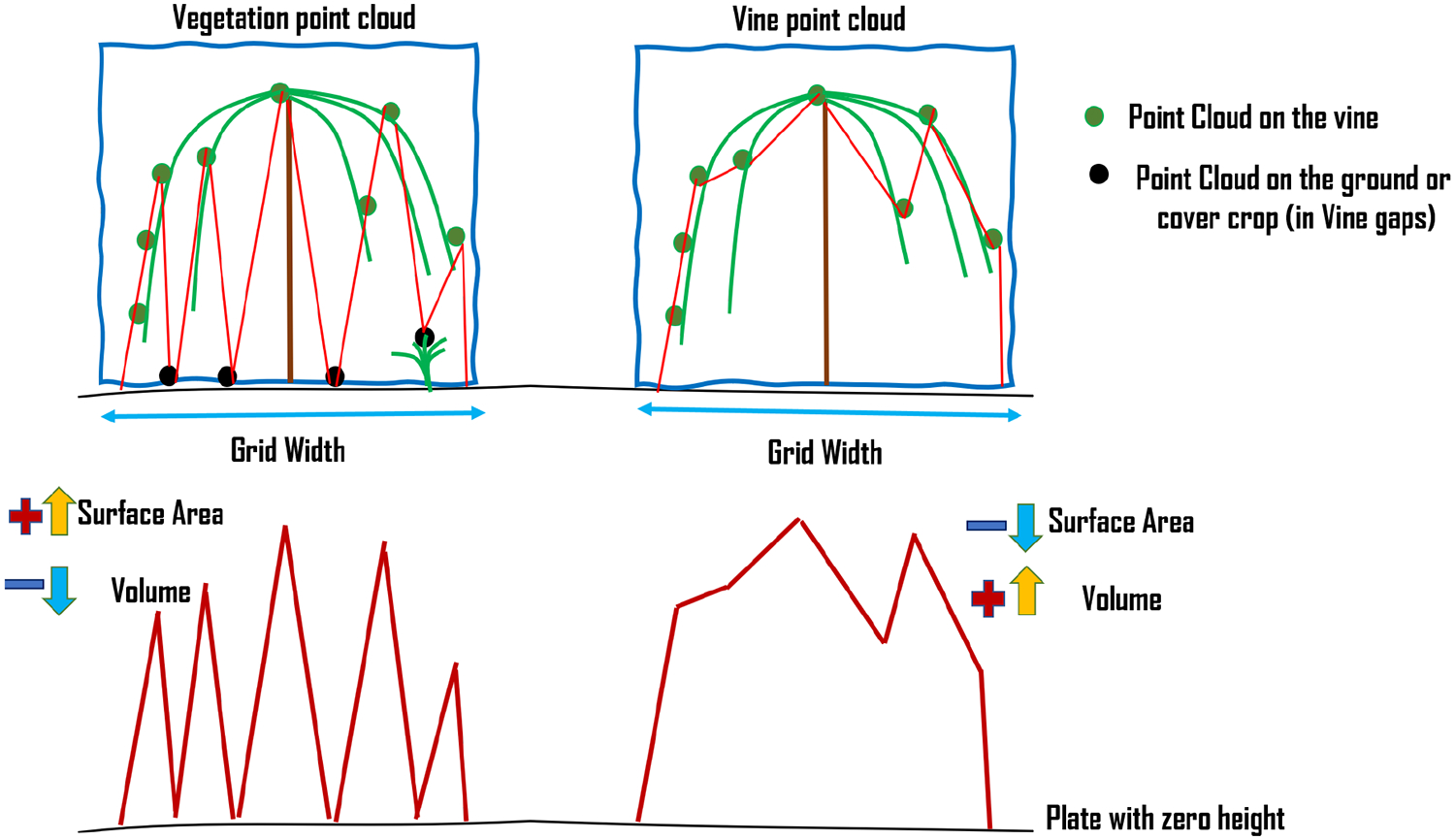
Impact of filtering z < 0.5 m on the vegetation/canopy volume and surface area.

As illustrated in Figure 13, volume and surface area are separately calculated for vegetation and vine canopy points due to the presence of grass cover crop. In terms of volume and surface area estimation, the main difference between vegetation and vine canopy is that the vegetation TIN file is created based on all non-zero heights, while, in the vine TIN file, points with height less than 0.5 m are excluded (Figure 7). As shown in Figure 14, this exclusion leads to increasing vegetation surface area and decreasing vegetation volume compared to structural vine information if gaps inside the vines are detected in the photogrammetry process (Figure A2c vs. Figure A2d and Figure A2a vs. Figure A2b).

### 3.2. Computation Time of VSSIXA

Although VSSIXA can precisely estimate structural information from point cloud data, the speed of the computational process is relatively slow due to the massive calculations needed to append spectral information into point cloud data and create TIN files. We used a relatively fast computer with a 2-terabyte Solid-state drive (SSD), 12 cores, 24 logical processors, and 128 gigabytes of Double Data Rate 4 (DDR4) RAM to execute VSSIXA over the study area. However, for each 3.6-m grid, both VSSIXA-I and VSSIXA-II require ~40 s to extract and store spectral-structural information. The study area contains ~77,000 grids. Therefore, each flight takes 35 days (77,000 × 40/3600/24) to be processed by VSSIXA. The 2015 July point cloud was processed by four fast computers to decrease the total running time to two weeks. Due to the long computational time of VSSIXA, spectral-structural information of other flights was extracted for footprints of the eddy covariance instrument and in situ LAI domains. It is possible that parallelization can enhance VSSIXA performance, but further investigation is needed.

### 3.3. In-Situ LAI versus VSSIXA Outputs

To evaluate the relationship between VSSIXA outputs and in situ LAI measurements, first the footprint of the LICOR-2200C must be defined. According to [14], it was assumed that the LICOR-2200C is measuring LAI in a rectangle 1 m wide and 3 m long. However, the smallest valid resolution of the TSEB model for the study area is a 3.6-m grid (square), which means that all required inputs for the TSEB model must be set to 3.6-m grids. Due to inconsistency between the LICOR-2200C footprint and the TSEB model resolution and its unknown impact on the LAI map, VSSIXA is executed for both rectangular and square buffers around LAI measurements (Figure 6).

To assess the performance of VSSIXA-I and VSSIXA-II, and particularly the importance of precise ground points (ground LiDAR dataset), spectral and structural information of the vegetation and canopy are computed by both versions of VSSIXA (VSSIXA-I and VSSIXA-II) and for both rectangular and square buffers (Figure 6). The relationship between in situ LAIs and VSSIXA outputs based on *R*^2^ are illustrated in Table 3.

Table 3 shows *R*^2^ calculated between in situ LAI and VSSIXA outputs. In general, results showed that structural information is more correlated to LAI compared to UAV spectral information, and among all the structural-spectral information extracted by VSSIXA, nine parameters had stronger correlation with LAI: NDVI, Tr, *N*_*v*_, *Volume*_*v*_, *SArea*_*v*_, *Area*_*v*_, *Volume*_*vc*_, *SArea*_*vc*_, *Area*_*vc*_. According to the definition of LAI [total one-sided leaf area per unit ground surface area], the strongest correlation was expected to be between LAI and surface areas (*SArea*_*v*_ and *SArea*_*vc*_). Table 3 shows that, in most cases, the strongest correlations associated with surface areas. The magnitude of those correlations was up to 44% in terms of *R*^2^, whereas vine canopy volume and vegetation volume (*Volume*_*v*_ and *Volume*_*vc*_) have reached 51%. Except for the June 2015 flight, no significant correlation was noted between vegetation and canopy height (*h*_*v*_ and *h*_*vc*_) versus LAI. Projected areas (*Area*_*v*_ and *Area*_*vc*_) are related to fractional cover, and fractional cover is nonlinearly related to LAI. Table 3 shows that the correlation between projected area, specifically vine canopy projected areas (*Area*_*vc*_), and LAI is comparable with volume information. In addition, results revealed that NIR and Tr bands, and consequently indices utilizing these two bands, have the potential to be used for LAI prediction for late vine growth stage.

Concerning the buffer shapes (square or rectangular) around LAI measurements, Table 3 shows that the correlation between spectral information and LAI is insensitive to the shape of the buffer, which means that the average values of spectral information in both grid sizes are close to each other. In contrast, changing the buffer grids from the rectangular to the square shape, in most cases, improves *R*^2^. For example, in the June 2015 flight at the Landsat time overpass (10:43 a.m.), *Volume*_*v*_, *Volume*_*vc*_, and *SArea*_*vc*_’s *R*^2^ doubled (16% to 38%, 15% to 36%, and 11% to 25%, respectively). Although the improvement in *R*^2^ with buffer shape change is not significant, VSSIXA-I’s performance appears to be more sensitive to the buffer shape. When VSSIXA-I is used along with the square buffer, the chance of ground point detection increases and may lead to improvements in the estimation of structural information. In other words, if narrower buffers are occupied by vine, VSSIXA-I considers the lowest height values of the vine canopy as the ground points, leading to a bias in structural information, particularly in vegetation and vine volumes (*Volume*_*v*_ and *Volume*_*vc*_).

Regarding VSSIXA-I and VSSIXA-II performances, since VSSIXA-II takes advantage of a more accurate ground point dataset (LiDAR ground data), it provides a more accurate estimation of structural information. Except for the May 2016 flight, volumes, surface areas, and projected surface areas calculated by VSSIXA-II are more correlated to in situ LAI. Our preliminary investigation on 2016 ground points extracted by the point cloud and LiDAR data shows that ground point cloud data are significantly lower than LiDAR data, which could be due to generating the point cloud using only two bands (R and NIR) compared to 2014 and 2015 point cloud data generated by four bands (R, G, B, and NIR).

### 3.4. Modeled LAI with Machine Learning Algorithms

**Figure 15.**
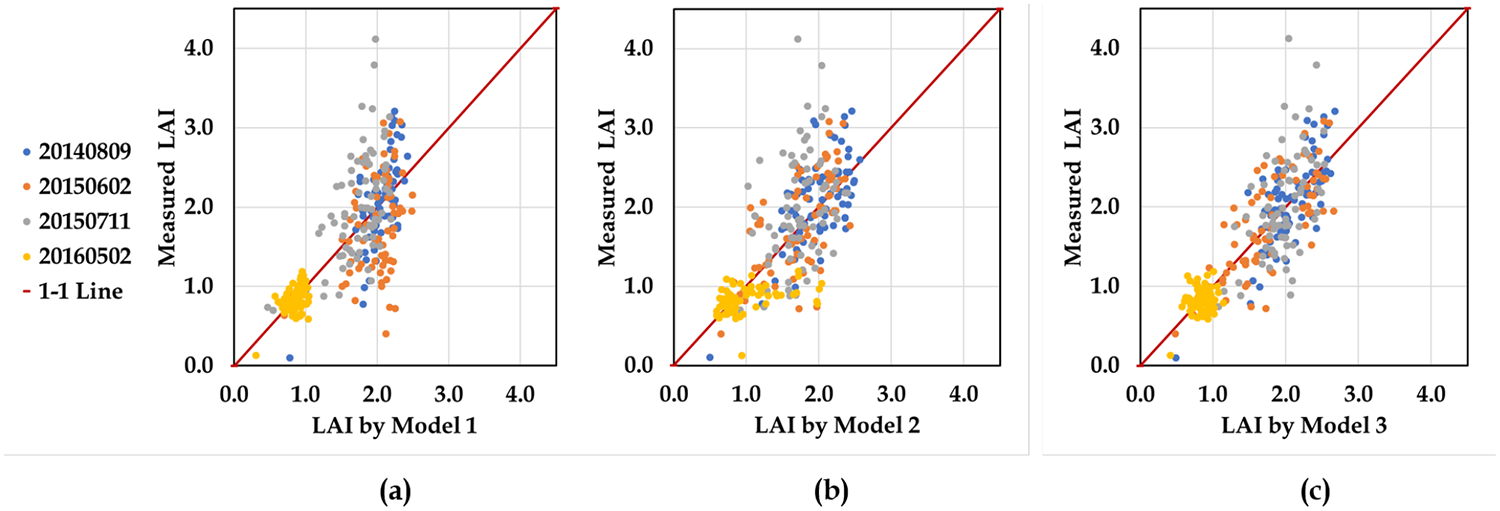
In situ LAI measurements versus modeled LAIs by GP based on Model 1 (**a**), Model 2 (**b**), and Model 3 (**c**).

Although VSSIXA-II outputs with the square buffers, in general, show higher correlations in terms of *R*^2^, this statistical analysis shows that a simple linear regression model cannot lead to an accurate LAI model across different vine growth stages, and exploring the ability of sophisticated algorithms such as machine learning techniques becomes necessary in modeling LAI. Machine learning techniques are not as simple as the regression models, but they can explore both linear and nonlinear relationships between output and several inputs through training and testing procedures that minimize error functions. Here, GP is employed to model LAI, exploring linear and nonlinear fitting curves between VSSIXA-II outputs extracted in square buffer domains. To remove the dependency of GP LAI models to the grid size, structural information (such as canopy volume and surface area) was divided by the area of the square grid (3.6 × 3.6 m). To evaluate the importance of structural information in modeled LAI, three different scenarios were defined, including LAI models with only spectral information (Model 1), with only structural information (Model 2), and with both spectral and structural information (Model 3). According to Table 3, N, NDVI, Tr, *N*_*v*_, and *N*_*vc*_ are the main inputs in Model 1. In Model 2, *Volume*_*v*_, *SArea*_*v*_, *Area*_*v*_, *Volume*_*vc*_, *SArea*_*vc*_, and *Area*_*vc*_ are considered as the main descriptors for the LAI model. In Model 3, a combination of Model 1 and Model 2 inputs are used to train GP and create the LAI map. Figure 15 and Table 4 show the results of the LAI modeled by GP and ~310 LAI measurements in the 2014, 2015, and 2016 flights, except for those lacking NIR or R bands.

As shown in Figure 15 and Table 4, employing GP with both spectral and structural information (Model 3) can significantly increase the accuracy of modeled LAI up to 70% in terms of *R*^2^ and enhance the performance of the models from fair to good (RRMSE of Model 1 and Model 2 < 30% compared to RRMSE of Model 3 < 20%). Despite flight time and vine phenological stage, GP was able to produce a reliable model if both spectral and structural information are provided. Equations (13)–(15) show the relationship between inputs and outputs found by GP for Models 1, 2, and 3, respectively:

(13) $$LAI_{1}=5.85+17.37\times N\times N_{v}+0.85\times NDVI\times Tr-0.52\times Tr-8.51\times N_{vc}^{2}-14.96\times NDVI^{2},$$

(14) $$LAI_{2}=0.47+2.39\times Area_{vc}-2.29\times Area_{vc}\times Area_{v}^{0.41\times 43.07^{{\text{Volume}}_{v}}},$$

(15) $$LAI_{3}=2.69\times N\times {\text{Volume}}_{vc}+0.11\times Tr\times Area_{v}+-0.67\times \frac{Area_{v}}{N_{vc}}-0.38\times 1.54^{Tr}\times N^{2}\times NDVI^{26.92}\times \frac{N_{vc}^{4}}{{\text{Volume}}_{vc}}.$$

The unit of Tr in Equations (13)–(15) is Celsius degree, and the unit of structural parameters is *m* as they are divided by the area of the square grids (*m*^3^/*m*^2^).

### 3.5. TSEB-2T Model versus Eddy Covariance Measurements

To evaluate the importance of point cloud data on the TSEB model, three different scenarios are defined. In scenario 1 (the spectral-based scenario, S1), the LAI map is created with GP Model 1. Canopy height (*h*_*vc*_), fractional cover (*f*_*c*_), and canopy width (*w*_*c*_) are set to fixed values. In scenario 2 (the structural-based scenario, S2), GP Model 2 is used to create the LAI map. *h*_*vc*_, *f*_*c*_ (vine projected surface area/the grid area), and *w*_*c*_ maps (3.35*f*_*c*_ [67]) are estimated by VSSIXA outputs instead of the fixed values used in S1. In Scenario 3 (the spectral-structural-based scenario, S3), the LAI map is created using GP Model 3 and other TSEB inputs the same as S2 (Table 5). Considering these three scenarios, the results of the modeled flux components by TSEB (Rn, LE, H, and G) are compared with the surface energy balance measurements from the Eddy Covariance flux tower footprints.

**Figure A1.**
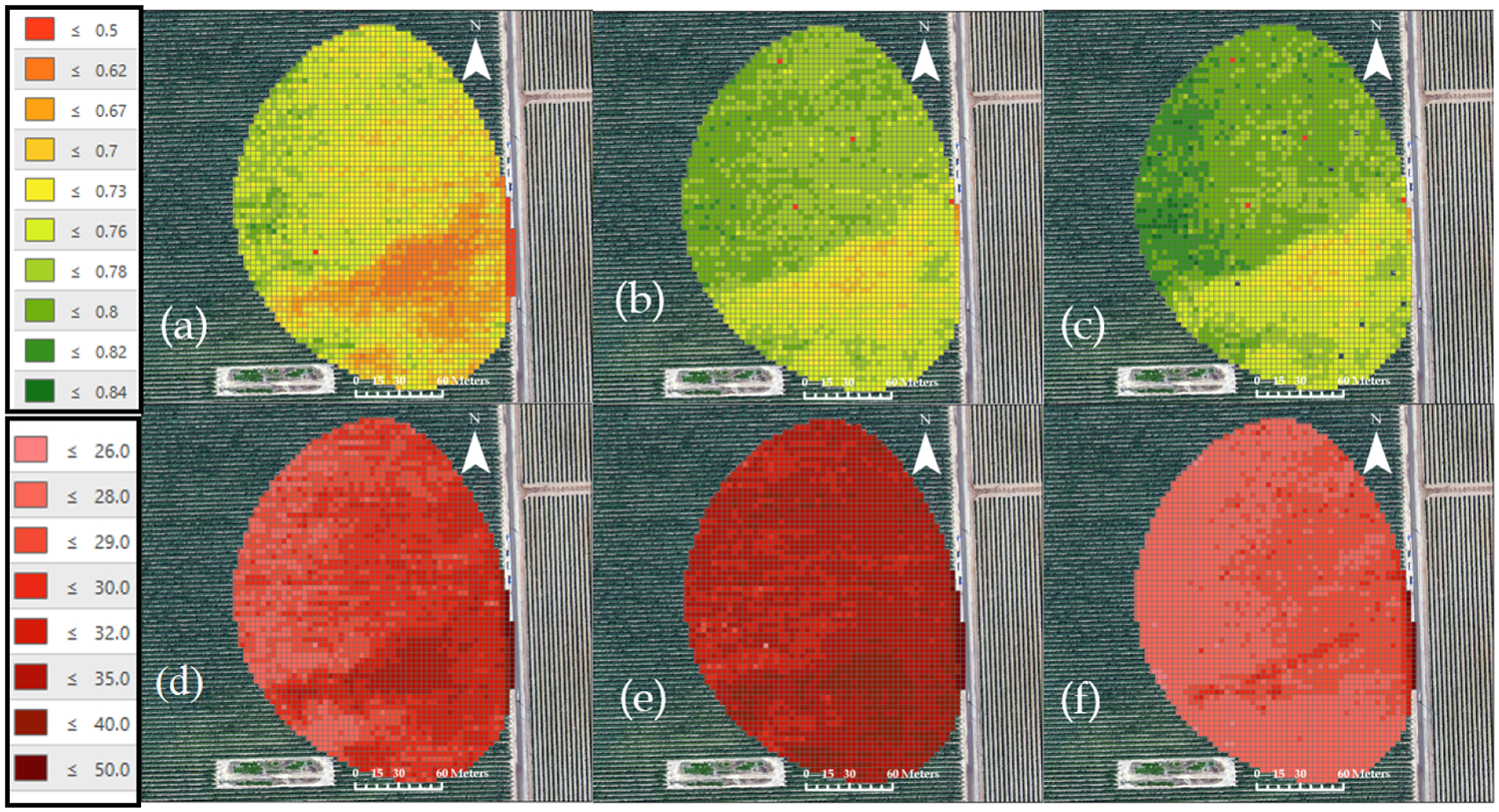
Examples of (**a**) NDVI, (**b**) *NDVI*_*V*_, (**c**) *NDVI*_*c*_, (**d**) *Tr*, (**e**) *Ts* and (**f**) *Tc* in centigrade calculated by VSSIXA-II for each 3.6 m grid of the northern flux tower foot print for July 2015 flight. Void cells are areas of missing data.

**Figure A2.**
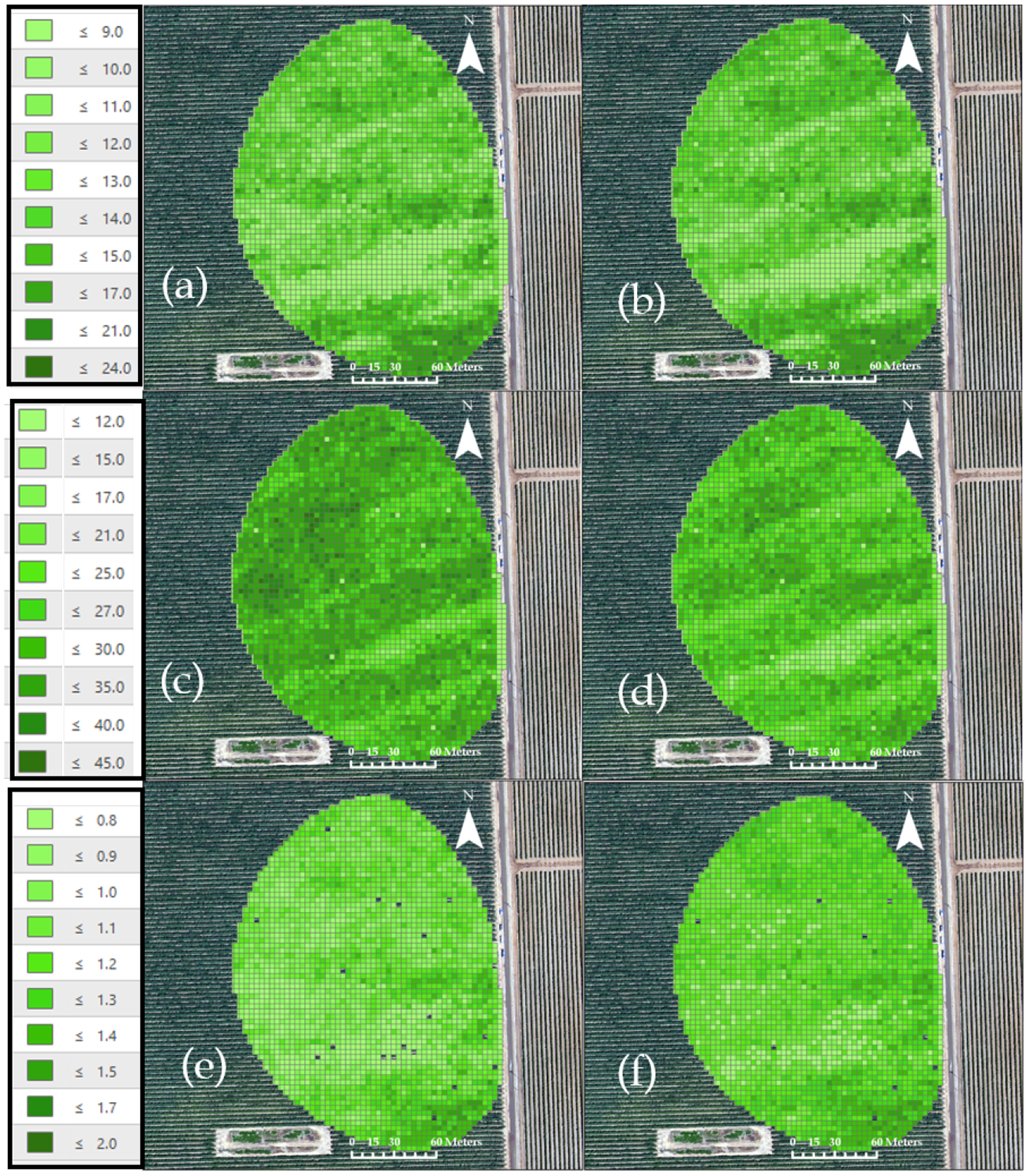
Examples of (**a**) *Volume*_*V*_, (**b**) *Volume*_*C*_ in *m*^3^, (**c**) *SArea*_*V*_, (**d**) *SArea*_*c*_ in *m*^2^, (**e**) *h*_*v*_ and (**f**) *h*_*vc*_ in *m* estimated by VSSIXA-II for each 3.6 m grid of the northern flux tower foot print for July 2015 flight. Void cells are areas of missing data.

To create LAI maps for each scenario at the TSEB resolution, VSIXXA-II with the square buffer is employed to extract spectral and structural information from the 2014, 2015, and 2016 flights. Next, an LAI map for each flight is created based on Models 1, 2 and 3. Due to the computation time of VSSIXA discussed in Section 3.2, VSSIXA-II is executed only for the flux tower footprints (see Figures A1 and A2). As shown in Figure 1, the study area includes two flux towers, the footprint of each tower contains ~ 2500 3.6-m grids that requires ~ 24 h (2500 × 40 s/3600 s) to process (Figures A1 and A2). The footprint of the flux tower is produced using a method presented by [91].

**Figure 16.**
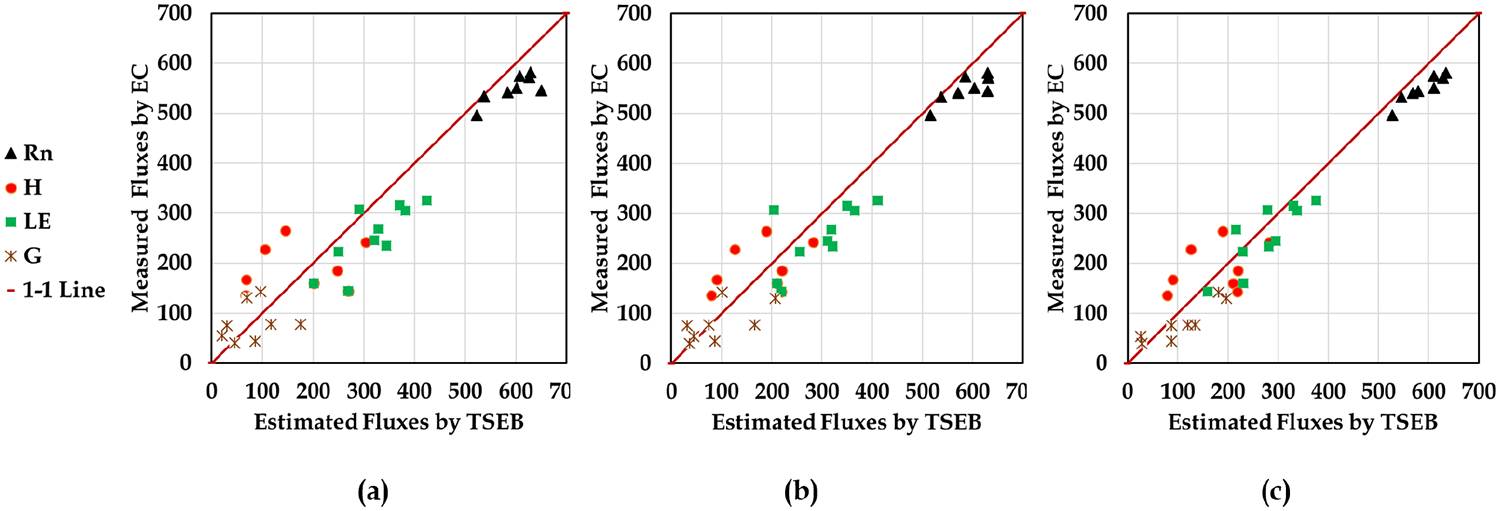
Scatterplot of observed vs. predicted fluxes using the different scenarios. (**a**) S1: LAI Model 1 and fixed values for *h*_*vc*_, *f*_*c*_, *w*_*c*_ (**b**) S2: LAI Model 2 with the map of *h*_*vc*_, *f*_*c*_, *w*_*c*_ (**c**) S3: LAI Model 3 with the map of *h*_*vc*_, *f*_*c*_, *w*_*c*_.

The results of the TSEB model compared to the eddy covaraince measurements are shown in Figure 16 and Table 6.

Figure 16 shows the agreement between TSEB model outputs versus eddy covariance measurements for each scenarios. Each subplot contains 32 pairs of estimated and observed energy fluxes (4 flights × 2 eddy covariance × 4 fluxes). From Figure 16, the agreement between modeled and observed fluxes improves going from using as LAI input GP Model 1 (S1) to GP Model 3 (S3), with the most significant improvement using S3 versus S1. Since differences between the performance of TSEB using GP Model 1 versus GP Model 2 for estimating LAI was not significant (Figure 16 and Table 6), it is likely that the improvement is mainly attributed to the use of a spatially-distributed map of the fractional cover, canopy height, and canopy width instead of using a fixed value. Using the spatially-distributed maps of the fractional cover, canopy height, and canopy width appears to have the largest effect on modeled H, with marginal impact on Rn, G, and LE. Comparing TSEB model results using S3 versus S2 and S1 reveals how a more accurate LAI map can affect the TSEB model output, particularly H and LE. The differences between TSEB output using S3 versus S2 illustrates the impact of the LAI maps, as the only difference between these two scenarios is related to the estimated LAI (*LAI*_2_ via Equation (13) and *LAI*_3_ via Equation (14)). According to Table 6, using GP Model 3 estimates of LAI in the TSEB model yields the best agreement with the observed H and LE fluxes. In terms of the RRMSE statistic for accuracy or performance of the TSEB model changes from “fair” to “good” rating for LE and “poor” to “fair” rating for H (i.e., poor rating is if RRMSE > 30%, fair rating if RRMSE < 30%, and a good rating if RRMSE < 20%). For Rn, all three GP model inputs of LAI produce an RRMSE value with “excellent” accuracy rating. On the other hand, the RRMSE value for G using all three GP models results in a “poor” rating. This “poor” performance is due in part to the assumption that G is a simple fraction of modeled soil net radiation (e.g., *G* = 0.30*Rn*_*s*_), but also the large spatial and temporal variability in measured G due to a nonuniform vine canopy cover [92] and the fact that the source area/flux footprint contributing to the tower fluxes and the area used in aggregating the TSEB model flux output is much greater than the sampling area used for the flux tower.

## 4. Discussion

In this study, a new algorithm, called VSSIXA, is developed to extract canopy spectral and structural information from multi-spectral UAV imagery and point cloud data. Although the computation time of VSSIXA is long (40 s for each 3.6-m grid), several aspects of this algorithm make it an efficient tool for improving remote sensing-based ET models, particularly the TSEB model. First, VSSIXA is able to separately extract vine canopy and cover crop canopy spectral and structural information, which cannot be achieved with solely spectral information. In other words, at the early phenological stage of the vine (April, May), when the presence of the cover crop is dominant, the spectral-based analysis cannot assign a unique class for vine and cover crop classes separately as their spectral responses are similar to one another. However, the structural information, particularly canopy height, can be an efficient measure for separation. This feature of VSSIXA can be useful for partitioning total flux into vine and interrow flux. Second, although vegetation indices (such as NDVI) are popular and well-known inputs for modeling LAI, these indices by themselves cannot fully describe the variability in LAI when the amount of active cover crop in the inter row is significant [67]. Therefore, 3D structural metrics can be used as other sources of information to derive spatial maps of LAI. The dominancy of the cover crop is more pronounced in the flights in May 2016 in which the active cover crop was present. In addition, several studies have indicated that satellite or UAV-derived LAI solely based on VIs may lead to the saturation situation that occurs within the relationship between VIs and LAI for well-developed canopies [6,10,11,13]. The saturation issue resulted from modeling a non-scaled parameter, namely LAI using scaled parameters such as VIs. However, as VSSIXA computed non-scaled structural metrics such as canopy height, surface area, and volume, the saturation issue does not occur in LAI estimated by Model 2 and Model 3, whereas most LAI values estimated by Model 1 ranged between 1 and 2 (Figure 15). Third, this study showed that, compared to using fixed-values, spatially-distributed structural metrics such as *hvc*, *f*_*c*_, and *w*_*c*_ can be more effective. However, a question may arise on how canopy structural properties can be re-generated or integrated into satellite imageries for estimation of daily canopy properties when no point cloud data exist for that coarse of pixel resolution or even for other dates. One approach is to fit empirical curves between in situ LAI values collected during different canopy phenological stages (bloom to harvest, Table 2) and structural information estimated by VSSIXA. Next, Landsat LAI obtained by fusing the MODIS LAI (MCD15A3H) product and Landsat surface reflectance [93,94] are trained with upscale structural canopy parameters (e.g., Landsat LAI vs. *hvc* at 30-m resolution). Ultimately, for each of the Landsat LAI products, spatially-distributed maps of canopy structural information at the satellite scale can be estimated based on satellite LAI products [95].

Although sensitivity analysis of canopy 3D metrics in remote sensing-based ET models, and particularly the TSEB model, require a further investigation, the authors in [96] performed a sensitivity analysis of the vegetation structural information (hc, LAI, fc, etc.) that is used in estimating soil resistance to heat transfer in sparse semiarid stands. Their results showed that the turbulent bulk heat transfer model for the sensible heat flux was sensitive to variations in crop height. The authors in [97]) conducted a simple model sensitivity analysis of TSEB to LAI and found that a variation on the LAI value of 30% would increase the final TSEB model error on a range of 4% and 7%. Thus, an error in LAI could significantly impact the accuracy of ET [98], which is compatible with the results presented in this study (decreasing LE from 72 (S2) to 39 (s3) in terms of RMSE). Generally, in the TSEB model, LAI is a key input for partitioning Tr into Ts and Tc and canopy and soil net radiation.

In TSEB-2T, the selection criterion for determining bare soil/cover crop stubble NDVI is based on the empirical relationship between NDVI and LAI [67]. In other words, *NDVI*_*S*_ in Figure 13 corresponds to the extrapolation of the NDVI-LAI curve for *LAI* = 0. Moreover, the spatial map of LAI is an input in the canopy radiative transfer model [87] to estimate soil and canopy net radiation (Equations (3)–(6)). Therefore, the partitioning of Rn between *Rn*_*s*_ and *Rn*_*c*_ is controlled by the LAI estimates. These equations (Equations (3) and (4)) indicate how and why the temporal trend in transpiration of the canopy (*LE*_*C*_) over LE follows the temporal variation in LAI [99]. In addition, LAI is inversely related to the the boundary layer resistance of the canopy of leaves (Equation (16)):

(16) $$R_{x}=\frac{C}{LAI}\times \left(\frac{l_{w}}{U_{d_{0}+Z_{0m}}}\right),$$

in which *d*_0_ is the zero-plane displacement height, and *z*_0*M*_ is the roughness length for momentum. *C* is assumed to be $90\frac{s^{\frac{1}{2}}}{m}$, and *l*_*w*_ is the average leaf width (m). Equation (16) indicates that overestimation of LAI leads to underestimation of *R*_*x*_ then overestimation of *H*_*c*_ and possibly an overestimation of *H* assuming a relatively small change in *H*_*s*_ (*H* = *H*_*s*_ + *H*_*c*_). As LE is calculated as a residual term of the land surface energy balance (*LE* = *R*_*n*_ − *G* − *H*), a lower *R*_*x*_ likely yields lower LE, assuming *R*_*n*_ and *G* are not highly sensitive to LAI.

In addition to relating LAI to NDVI thresholds of vegetation and bare soil/cover crop stubble, partitioning Rn into *Rn*_*s*_ and *Rn*_*c*_ and the boundary layer resistance of the canopy in the TSEB model, LAI is used to indirectly (through the partitioning of Rn into *Rn*_*s*_ and *Rn*_*c*_) estimate G via the expression *G* = 0.3*Rn*_*s*_. This resulted in estimated G from TSEB to be in relatively poor agreement with observed G (see Table 6). However, modifications to this simple expression have been proposed (Nieto et al. [67]), which considers empirically the effect of the cover crop on G.

## 5. Conclusions

This paper explored the utility of incorporating UAV point cloud products into the remote sensing-based TSEB model. A new algorithm called VSSIXA in Python and ArcGIS Pro was developed to extract both spectral and structural information for a vineyard. VSSIXA is developed in two modes, VSSIXA-I and VSSIXA-II. VSSIXA-I only requires point cloud data to calculate vegetation structural information, while VSSIXA-II requires a precise and separate ground point data (e.g., LiDAR data). In this study, both versions of VSSIXA along with different buffer shapes around in situ LAI measurements are employed to create LAI maps. Three different estimates of LAI using Genetic Programming (GP) machine learning are considered to evaluate the impact of structural information for computing LAI. First, results indicated that VSSIXA-II with wider buffers is more efficient for calculating vegetation structural information. Among the three GP-based models for estimating LAI, Model scenario 1 (S1) and Model scenario 2 (S2), which require only spectral and structural information, respectively, had similar performance, while Model scenario 3 (S3), which takes advantage of both spectral and structural information, could estimate LAI with 70% accuracy in terms of *R*^2^.

To assess the impact of the structural information in modeling fluxes, the remote sensing-based TSEB model was applied using the three LAI modeling scenarios, S1–S3 and using fixed values versus a spatially-distributed map of canopy height, width, and fractional cover. The TSEB model output of the fluxes using derived soil and canopy temperatures (TSEB-2T), which avoids the need for the Priestley–Taylor assumption for canopy transpiration, are compared with eddy covariance flux tower measurements. Results indicated that significant improvement in the agreement of modeled output with the flux tower observations is achieved by using a reliable LAI map, more so than a map of spatially-distributed canopy structure parameters. The statistical results suggest that a more robust LAI map derived from both spectral and structural information can lead to significant improvement in TSEB model performance in estimating the turbulent fluxes H and LE. There was much less of an impact from the three different model estimates of LAI in TSEB output of *Rn* and G. In particular, the relatively poor performance rating given by the RRMSE statistic for G has to do with both the simple model assumption that G is a constant fraction of *Rn*_*s*_ and the significant spatial and temporal variation in individual G measurements observed by [92]. Improvements on this simple formulation for estimating G have been proposed by Nieto et al. [67].

## Figures and Tables

**Table 1.** Dates, times, cameras ^1^, and optical filters used to capture images with the UAV.

Date	UAV Flight Time (PDT)		UAV Elevation (agl) Meters	Bands		Cameras and Optical Filters		Spectral Response
	Lunch Time	Landing		RGB	NIR	Radiometric Response	MegaPixels	
9 August 2014	11:30 a.m.	11:50 a.m.	450	Cannon S95	Cannon S95 modified (Manufacturer NIR block filter removed)	8-bit	10	RGB: typical CMOS NIR: extended CMOS NIR Kodak Wratten 750 nm0020 LongPass filter
2 June 2015	11:21 am.	12:06 p.m.	450	Lumenera Lt65R Color	Lumenera Lt65R Monochrome	14-bit	9	RGB: typical CMOS NIR: Schneider 820 nm LongPass filter
11 July 2015	11:26 a.m.	12:00 p.m.	450	Lumenera Lt65R Color	Lumenera Lt65R Monochrome	14-bit	12	RGB: typical CMOS NIR: Schneider 820 nm LongPass filter
2 May 2016	12:53 p.m.	1:17 p.m.	450	Lumenera Lt65R Mono	Lumenera Lt65R Mono	14-bit	12	RGB: Landsat 8 Red Filter equivalent NIR: Landsat 8 NIR Filter equivalent

1 The use of trade, firm, or corporation names in this article is for the information and convenience of the reader. Such use does not constitute official endorsement or approval by the US Department of Agriculture or the Agricultural Research Service of any product or service to the exclusion of others that may be suitable.

**Table 2.** Dates, optical and thermal resolution, point cloud density and phenological stages of the vine and cover crop when the images were captured by the UAV.

Date	Optical Resolution	Thermal Resolution	Point Cloud Density (Point/m^2^)	Vine Phenological Stage	Phenological Stage of Cover Crop
9 August 2014	15 cm	60 cm	37	Veraison towards harvest	Mowed stubble
2 June 2015	10 cm	60 cm	118	Near veraison	Senescent
11 July 2015	10 cm	60 cm	108	Veraison	Mowed stubble
2 May 2016	10 cm	60 cm	120	Bloom to fruit set	Active/green

**Table 3.** *R*^2^ calculated between VSSIXA outputs and in situ LAI measurements for 2014, 2015, and 2016 UAV flights over Sierra Loma.

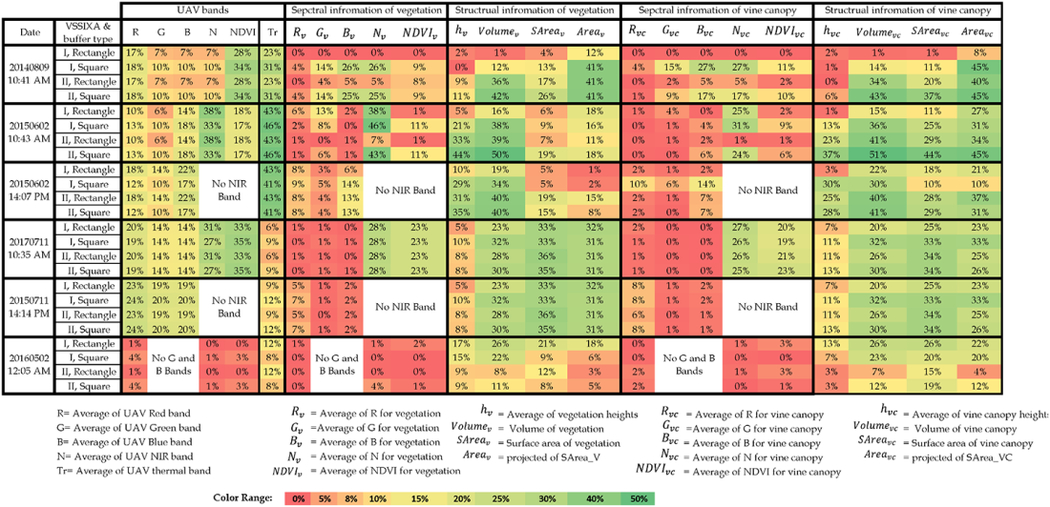

**Table 4.** Performance of the Models 1, 2 and 3.

Stats	Model 1	Model 2	Model 3
*R*^2^	0.56	0.54	0.70
MAE	0.35	0.37	0.30
RMSE	0.43	0.44	0.32
RRMS	25%	26%	19%

**Table 5.** TSEB Inputs for each scenario.

Scenario	LAI	*h*_*vc*_ (Canopy Height)	*f*_*c*_ (Fractional Cover)	*w*_*c*_ (Canopy Width)
S1: Spectral-based	GP Model 1	a fixed value	a fixed value	a fixed value
S2: Structural-based	GP Model 2	estimated by VSSIXA	estimated by VSSIXA	= 3.35 * *f*_*c*_
S3: Spectral-Structural-based	GP Model 3	estimated by VSSIXA	estimated by VSSIXA	= 3.35 * *f*_*c*_

**Table 6.** Performance of the TSEB model based on GP model estimate of LAI using model scenarios 1, 2, and 3 (S1, S2 and S3) for each energy flux component.

Variable	Scenario	MAE	RMSE	RRMSE
Rn	S1	46	53	10%
	S2	39	47	8%
	S3	39	42	8%
H	S1	87	93	49%
	S2	64	67	35%
	S3	35	40	21%
LE	S1	65	72	26%
	S2	65	69	25%
	S3	35	39	14%
G	S1	46	52	65%
	S2	38	49	61%
	S3	37	41	51%

## Abbreviations

The following abbreviations are used in this manuscript
UAV: Unmanned Aerial Vehicles
TSEB: Two-Source Energy Balance Model
VSSIXA: Vegetation Structural-Spectral Information eXtraction Algorithm
LAI: Leaf Area Index
GRAPEX: Grape Remote sensing Atmospheric Profile and Evapotranspiration eXperiment
VIs: Vegetation indices
R: Red
G: Green
B: Blue
NIR: Near-Infrared
NDVI: Normalized Difference Vegetation Index
DSM: Digital Surface Models
SfM: Structure from Motion
MVS: Multiview-Stereo
LiDAR: Light Detection and Ranging
CSM: Crop Surface Model
GCP: Ground Control Points
CHM: Canopy Height Model
DEM: Digital Elevation Model
DTM: Digital Terrain Model
*T_r_*: Radiometric Temperature
USU: Utah State University
IMU: Inertial Measurement Unit
VNIR: Visible and Near-Infrared
*T_s_*: Soil Temperature
*T_c_*: Canopy Temperature
TIN: Triangulated Irregular Network
CSV: Comma-Separated Value
ANN: Artificial Neural Network
SVM: Support Vector Machine
GP: Genetic Programming
IOP: Intensive Observation Period
ASTER: Advanced Spaceborne Thermal Emission and Reflection Radiometer
agl: above ground level
ESRI: Environmental Systems Research Institute
USU: Utah State University
G-LiHT: Goddard’s LiDAR, Hyperspectral & Thermal Imager
IRGA: Infrared Gas Analyzer
GA: Genetic Algorithm
*S_n_*: Shortwave Radiation
*L_n_*: Longwave Radiation
*Ln_c_*: Canopy Net Longwave Radiation
*Ln_s_*: Soil Net Longwave Radiation
*Sn_c_*: Canopy Net Shortwave Radiation
*Sn_s_*: Soil Net Shortwave Radiation
*Rn_c_*: Canopy Net Radiation
*Rn_s_*: Soil Net Radiation
*G*: Soil Heat Flux
*H_c_*: Sensible Heat Flux for Canopy
*H_s_*: Sensible Heat Flux for Soil
*LE_c_*: Latent Heat Flux for Canopy
*LE_s_*: Latent Heat Flux for Soil
*R*2: Coefficient of Determination
MAE: Mean Absolute Error
RMSE: Root Mean Square Error
RRMSE: Relative Root Mean Square Error
RTK: Real-Time Kinematic
*R_v_*: Average of R for Vegetation
*G_v_*: Average of G for Vegetation
*B_v_*: Average of B for Vegetation
*N_v_*: Average of N for Vegetation
*NDVI_v_*: Average of NDVI for Vegetation
*h_v_*: Average of Vegetation Heights
*Volume_v_*: Volume of Vegetation
*SArea_v_*: Surface area of Vegetation
*Area_v_*: Projected of *SArea*_*v*_
*R_vc_*: Average of R for Vine Canopy
*G_vc_*: Average of G for Vine Canopy
*B_vc_*: Average of B for Vine Canopy
*N_vc_*: Average of N for Vine Canopy
*NDVI_vc_*: Average of NDVI for Vine Canopy
*h_vc_*: Average of Vine Canopy Height
*Volume_vc_*: Volume of Vine Canopy
*SArea_vc_*: Surface Area of Vine Canopy
*Area_vc_*: Projected of *SArea*_*vc*_
*f_c_*: Fractional Cover
*w_c_*: Canopy Width
*S*1: Scenario 1
*S*2: Scenario 2
*S*3: Scenario 3

## References

[1] Colaizzi PD; Kustas WP; Anderson MC; Agam N; Tolk JA; Evett SR; Howell TA; Gowda PH; O’Shaughnessy SA Two-source energy balance model estimates of evapotranspiration using component and composite surface temperatures. Adv. Water Resour 2012, 50, 134–151. doi:10.1016/j.advwatres.2012.06.004.

[2] Tang R; Li ZL; Jia Y; Li C; Sun X; Kustas WP; Anderson MC An intercomparison of three remote sensing-based energy balance models using Large Aperture Scintillometer measurements over a wheat–corn production region. Remote Sens. Environ 2011, 115, 3187–3202. doi:10.1016/j.rse.2011.07.004.

[3] Timmermans WJ; Kustas WP; Anderson MC; French AN An intercomparison of the Surface Energy Balance Algorithm for Land (SEBAL) and the Two-Source Energy Balance (TSEB) modeling schemes. Remote Sens. Environ 2007, 108, 369–384. doi:10.1016/j.rse.2006.11.028.

[4] Anderson MC; Norman JM; Kustas WP; Li F; Prueger JH; Mecikalski JR Effects of Vegetation Clumping on Two–Source Model Estimates of Surface Energy Fluxes from an Agricultural Landscape during SMACEX. J. Hydrometeorol 2005, 6, 892–909. doi:10.1175/JHM465.1.

[5] Norman J; Kustas W; Humes K Source approach for estimating soil and vegetation energy fluxes in observations of directional radiometric surface temperature. Agric. For. Meteorol 1995, 77, 263–293. doi:10.1016/0168-1923(95)02265-Y.

[6] Aboutalebi M; Torres-Rua AF; Allen N Multispectral remote sensing for yield estimation using high-resolution imagery from an unmanned aerial vehicle In Proceedings of the Autonomous Air and Ground Sensing Systems for Agricultural Optimization and Phenotyping III, Orlando, FL, USA, 15–19 4 2018; Volume 10664. doi:10.1117/12.2305888.

[7] Kumar L; Schmidt K; Dury S; Skidmore A Imaging Spectrometry and Vegetation Science In Imaging Spectrometry: Basic Principles and Prospective Applications; Meer F.D.v.d., Jong SMD, Eds.; Springer: Dordrecht, The Netherlands, 2001; pp. 111–155. doi:10.1007/978-0-306-47578-8_5.

[8] Xue J; Su B Significant Remote Sensing Vegetation Indices: A Review of Developments and Applications. J. Sensors 2017, 2017. doi:10.1155/2017/1353691.

[9] Sun L; Gao F; Anderson MC; Kustas WP; Alsina MM; Sanchez L; Sams B; McKee L; Dulaney W; White WA; Daily Mapping of 30 m LAI and NDVI for Grape Yield Prediction in California Vineyards. Remote Sens 2017, 9, 317. doi:10.3390/rs9040317.

[10] Asrar G; Fuchs M; Kanemasu ET; Hatfield JL Estimating Absorbed Photosynthetic Radiation and Leaf Area Index from Spectral Reflectance in Wheat. Agron. J 1989, 76, 300–306. doi:10.2134/agronj1984.00021962007600020029x.

[11] Serrano L; Filella I; Peñuelas J Remote sensing of biomass and yield of winter wheat under different nitrogen supplies. Crop. Sci 2000, 40, 723–731.

[12] Bendig J; Yu K; Aasen H; Bolten A; Bennertz S; Broscheit J; Gnyp ML; Bareth G Combining UAV-based plant height from crop surface models, visible, and near infrared vegetation indices for biomass monitoring in barley. Int. J. Appl. Earth Obs. Geoinf 2015, 39, 79–87. doi:10.1016/j.jag.2015.02.012.

[13] Diarra A; Jarlan L; Er-Raki S; Page ML; Aouade G; Tavernier A; Boulet G; Ezzahar J; Merlin O; Khabba S Performance of the two-source energy budget (TSEB) model for the monitoring of evapotranspiration over irrigated annual crops in North Africa. Agric. Water Manag 2017, 193, 71–88. doi:10.1016/j.agwat.2017.08.007.

[14] White WA; Alsina MM; Nieto H; McKee LG; Gao F; Kustas WP Determining a robust indirect measurement of leaf area index in California vineyards for validating remote sensing-based retrievals. Irrig. Sci 2018, 37, 269–280.

[15] Zarco-Tejada P; Diaz-Varela R; Angileri V; Loudjani P Tree height quantification using very high resolution imagery acquired from an unmanned aerial vehicle (UAV) and automatic 3D photo-reconstruction methods. Eur. J. Agron 2014, 55, 89–99. doi:10.1016/j.eja.2014.01.004.

[16] Du M; Noguchi N Monitoring of Wheat Growth Status and Mapping of Wheat Yield’s within-Field Spatial Variations Using Color Images Acquired from UAV-camera System. Remote Sens 2017, 9, 289. doi:10.3390/rs9030289.

[17] Zermas D; Teng D; Stanitsas P; Bazakos M; Kaiser D; Morellas V; Mulla D; Papanikolopoulos N Automation solutions for the evaluation of plant health in corn fields In Proceedings of the 2015 IEEE/RSJ International Conference on Intelligent Robots and Systems (IROS), Hamburg, Germany, 28 September–2 October 2015; pp. 6521–6527. doi:10.1109/IROS.2015.7354309.

[18] Santesteban L; Gennaro SD; Herrero-Langreo A; Miranda C; Royo J; Matese A High-resolution UAV-based thermal imaging to estimate the instantaneous and seasonal variability of plant water status within a vineyard. Agric. Water Manag 2017, 183, 49–59. doi:10.1016/j.agwat.2016.08.026.

[19] Jiménez-Bello MA; Royuela A; Manzano J; Zarco-Tejada PJ; Intrigliolo D Assessment of drip irrigation sub-units using airborne thermal imagery acquired with an Unmanned Aerial Vehicle (UAV) In Precision Agriculture ‘13; Stafford JV, Ed.; Wageningen Academic Publishers: Wageningen, The Netherlands, 2013; pp. 705–711.

[20] Holman FH; Riche AB; Michalski A; Castle M; Wooster MJ; Hawkesford MJ High Throughput Field Phenotyping of Wheat Plant Height and Growth Rate in Field Plot Trials Using UAV Based Remote Sensing. Remote Sens 2016, 8, 1031. doi:10.3390/rs8121031.

[21] Vanegas F; Bratanov D; Powell K; Weiss J; Gonzalez F A Novel Methodology for Improving Plant Pest Surveillance in Vineyards and Crops Using UAV-Based Hyperspectral and Spatial Data. Sensors 2018, 18, 260. doi:10.3390/s18010260. PMC5795822 29342101

[22] Rasmussen J; Nielsen J; Garcia-Ruiz F; Christensen S; Streibig JC Potential uses of small unmanned aircraft systems (UAS) in weed research. Weed Res 2013, 53, 242–248. doi:10.1111/wre.12026.

[23] Rokhmana CA The Potential of UAV-based Remote Sensing for Supporting Precision Agriculture in Indonesia. Procedia Environ. Sci 2015, 24, 245–253. doi:10.1016/j.proenv.2015.03.032.

[24] Comba L; Biglia A; Aimonino DR; Gay P Unsupervised detection of vineyards by 3D point-cloud UAV photogrammetry for precision agriculture. Comput. Electron. Agric 2018, 155, 84–95. doi:10.1016/j.compag.2018.10.005.

[25] Fraser RH; Olthof I; Lantz TC; Schmitt C UAV photogrammetry for mapping vegetation in the low-Arctic. Arct. Sci 2016, 2, 79–102. doi:10.1139/as-2016-0008.

[26] Thiel C; Schmullius C Comparison of UAV photograph-based and airborne lidar-based point clouds over forest from a forestry application perspective. Int. J. Remote Sens 2017, 38, 2411–2426. doi:10.1080/01431161.2016.1225181.

[27] Yilmaz V; Konakoglu B; Serifoglu C; Gungor O; Gökalp E Image classification-based ground filtering of point clouds extracted from UAV-based aerial photos. Geocarto Int 2018, 33, 310–320. doi:10.1080/10106049.2016.1250825.

[28] Hu X; Zhang Z; Duan Y; Zhang Y; Zhu J; Long H Lidar Photogrammetry and Its Data Organization. ISPRS-Int. Arch. Photogramm. Remote Sens. Spat. Inf. Sci 2011, XXXVIII-5/W12, 181–184. doi:10.5194/isprsarchives-XXXVIII-5-W12-181-2011.

[29] Küng O; Strecha C; Beyeler A; Zufferey JC; Floreano D; Fua P; Gervaix F The accuracy of automatic photogrammetric techniques on ultra-light UAV imagery. Int. Arch. Photogramm. Remote Sens. Spat. Inf. Sci.-ISPRS Arch 2011, 38, 125–130.

[30] Rock G; Ries JB; Udelhoven T Sensitivity Analysis of Uav-Photogrammetry for Creating Digital Elevation Models (DEM). ISPRS-Int. Arch. Photogramm. Remote Sens. Spat. Inf. Sci 2011, XXXVIII-1/C22, 69–73. doi:10.5194/isprsarchives-XXXVIII-1-C22-69-2011.

[31] Amrullah C; Suwardhi D; Meilano I Product Accuracy Effect of Oblique and Vertical Non-Metric Digital Camera Utilization in Uav-Photogrammetry to Determine Fault Plane. ISPRS Ann. Photogramm. Remote Sens. Spat. Inf. Sci 2016, III-6, 41–48. doi:10.5194/isprs-annals-III-6-41-2016.

[32] Mesas-Carrascosa FJ; Notario García MD; Meroño de Larriva JE; García-Ferrer A An Analysis of the Influence of Flight Parameters in the Generation of Unmanned Aerial Vehicle (UAV) Orthomosaicks to Survey Archaeological Areas. Sensors 2016, 16, 1838. doi:10.3390/s16111838. PMC5134497 27809293

[33] Dandois JP; Olano M; Ellis EC Optimal Altitude, Overlap, and Weather Conditions for Computer Vision UAV Estimates of Forest Structure. Remote Sens 2015, 7, 13895–13920. doi:10.3390/rs71013895.

[34] Verhoeven G Taking computer vision aloft—Archaeological three-dimensional reconstructions from aerial photographs with photoscan. Archaeol. Prospect 2011, 18, 67–73. doi:10.1002/arp.399.

[35] Tahar KN; Ahmad A An Evaluation on Fixed Wing and Multi-Rotor UAV Images Using Photogrammetric Image Processing. Int. J. Comput. Electr. Autom. Control. Inf. Eng 2013, 7, 48–52.

[36] Jaud M; Passot S; Le Bivic R; Delacourt C; Grandjean P; Le Dantec N Assessing the Accuracy of High Resolution Digital Surface Models Computed by PhotoScan® and MicMac® in Sub-Optimal Survey Conditions. Remote Sens 2016, 8, 465. doi:10.3390/rs8060465.

[37] Martínez-Carricondo P; Agüera-Vega F; Carvajal-Ramírez F; Mesas-Carrascosa FJ; García-Ferrer A; Pérez-Porras FJ Assessment of UAV-photogrammetric mapping accuracy based on variation of ground control points. Int. J. Appl. Earth Obs. Geoinf 2018, 72, 1–10. doi:10.1016/j.jag.2018.05.015.

[38] Aboutalebi M; Torres-Rua AF; McKee M; Kustas W; Nieto H; Coopmans C Validation of digital surface models (DSMs) retrieved from unmanned aerial vehicle (UAV) point clouds using geometrical information from shadows In Proceedings of the Autonomous Air and Ground Sensing Systems for Agricultural Optimization and Phenotyping IV, Baltimore, MD, USA, 14–18 4 2019; Volume 11008. doi:10.1117/12.2519694. PMC6662722 31359902

[39] Aboutalebi M; Torres-Rua AF; Kustas WP; Nieto H; Coopmans C; McKee M Assessment of different methods for shadow detection in high-resolution optical imagery and evaluation of shadow impact on calculation of NDVI, and evapotranspiration. Irrig. Sci 2019, 37, 407–429. doi:10.1007/s00271-018-0613-9. PMC6480411 31031514

[40] Garousi-Nejad I; Tarboton D; Aboutalebi M; Torres-Rua A Terrain Analysis Enhancements to the Height Above Nearest Drainage Flood Inundation Mapping Method. Water Resour. Res 2019, 55, 7983–8009

[41] Jensen JLR; Mathews AJ Assessment of Image-Based Point Cloud Products to Generate a Bare Earth Surface and Estimate Canopy Heights in a Woodland Ecosystem. Remote Sens 2016, 8, 50. doi:10.3390/rs8010050.

[42] Panagiotidis D; Abdollahnejad A; Surový P; Chiteculo V Determining Tree Height and Crown Diameter from High-resolution UAV Imagery. Int. J. Remote Sens 2017, 38, 2392–2410.

[43] Díaz-Varela RA; De la Rosa R; León L; Zarco-Tejada PJ High-Resolution Airborne UAV Imagery to Assess Olive Tree Crown Parameters Using 3D Photo Reconstruction: Application in Breeding Trials. Remote Sens 2015, 7, 4213–4232. doi:10.3390/rs70404213.

[44] Karpina M; Jarzabek-Rychard M; Tymków P; Borkowski A Uav-Based Automatic Tree Growth Measurement for Biomass Estimation. ISPRS-Int. Arch. Photogramm. Remote Sens. Spat. Inf. Sci 2016, XLI-B8, 685–688. doi:10.5194/isprs-archives-XLI-B8-685-2016.

[45] Kattenborn T; Sperlich M; Bataua K; Koch B Automatic Single Tree Detection in Plantations using UAV-based Photogrammetric Point clouds. ISPRS-Int. Arch. Photogramm. Remote Sens. Spat. Inf. Sci 2014, XL-3, 139–144. doi:10.5194/isprsarchives-XL-3-139-2014.

[46] Bendig J; Willkomm M; Tilly N; Gnyp ML; Bennertz S; Qiang C; Miao Y; Lenz-Wiedemann VIS; Bareth G Very high resolution crop surface models (CSMs) from UAV-based stereo images for rice growth monitoring In Northeast China. ISPRS-Int. Arch. Photogramm. Remote Sens. Spat. Inf. Sci 2013, XL-1/W2, 45–50. doi:10.5194/isprsarchives-XL-1-W2-45-2013.

[47] Bendig J; Bolten A; Bennertz S; Broscheit J; Eichfuss S; Bareth G Estimating Biomass of Barley Using Crop Surface Models (CSMs) Derived from UAV-Based RGB Imaging. Remote Sens 2014, 6, 10395–10412. doi:10.3390/rs61110395.

[48] Gitelson AA; Viña A; Arkebauer TJ; Rundquist DC; Keydan G; Leavitt B Remote estimation of leaf area index and green leaf biomass in maize canopies. Geophys. Res. Lett 2003, 30. doi:10.1029/2002GL016450.

[49] Honkavaara E; Kaivosoja J; Mäkynen J; Pellikka I; Pesonen L; Saari H; Salo H; Hakala T; Marklelin L; Rosnell T Hyperspectral Reflectance Signatures and Point Clouds for Precision Agriculture by Light Weight Uav Imaging System. ISPRS Ann. Photogramm. Remote Sens. Spat. Inf. Sci 2012, I-7, 353–358. doi:10.5194/isprsannals-I-7-353-2012.

[50] Duan T; Zheng B; Guo W; Ninomiya S; Guo Y; Chapman SC Comparison of ground cover estimates from experiment plots in cotton, sorghum and sugarcane based on images and ortho-mosaics captured by UAV. Funct. Plant Biol 2017, 44, 169–183. 10.1071/FP16123 32480555

[51] Calera A; Martínez C; Melia J A procedure for obtaining green plant cover: Relation to NDVI in a case study for barley. Int. J. Remote Sens 2001, 22, 3357–3362. doi:10.1080/01431160010020100.

[52] Matese A; Gennaro SFD; Berton A Assessment of a canopy height model (CHM) in a vineyard using UAV-based multispectral imaging. Int. J. Remote Sens 2017, 38, 2150–2160. doi:10.1080/01431161.2016.1226002.

[53] Mathews AJ; Jensen JLR Visualizing and Quantifying Vineyard Canopy LAI Using an Unmanned Aerial Vehicle (UAV) Collected High Density Structure from Motion Point Cloud. Remote Sens 2013, 5, 2164–2183. doi:10.3390/rs5052164.

[54] Weiss M; Baret F Using 3D Point Clouds Derived from UAV RGB Imagery to Describe Vineyard 3D Macro-Structure. Remote Sens 2017, 9, 111. doi:10.3390/rs9020111.

[55] Kustas WP; Anderson MC; Alfieri JG; Knipper K; Torres-Rua A; Parry CK; Nieto H; Agam N; White A; Gao F; The Grape Remote Sensing Atmospheric Profile and Evapotranspiration Experiment (GRAPEX) Bull. Amer. Meteorol. Soc 2018, 99, 1791–1812. doi:10.1175/BAMS-D-16-0244.1. PMC8022860 33828330

[56] Elarab M; Ticlavilca AM; Torres-Rua AF; Maslova I; McKee M Estimating chlorophyll with thermal and broadband multispectral high resolution imagery from an unmanned aerial system using relevance vector machines for precision agriculture. Int. J. Appl. Earth Obs. Geoinf 2015, 43, 32–42. doi:10.1016/j.jag.2015.03.017.

[57] Hassan-Esfahani L; Torres-Rua A; Jensen A; McKee M Assessment of Surface Soil Moisture Using High-Resolution Multi-Spectral Imagery and Artificial Neural Networks. Remote Sens 2015, 7, 2627–2646. doi:10.3390/rs70302627.

[58] Aggieair. https://uwrl.usu.edu/aggieair/ (accessed on 15 December 2019).

[59] Labsphere. https://www.labsphere.com (accessed on 15 December 2019).

[60] Neale CM; Crowther BG An airborne multispectral video/radiometer remote sensing system: Development and calibration. Remote Sens. Environ 1994, 49, 187–194. doi:10.1016/0034-4257(94)90014-0.

[61] Miura T; Huete A Performance of three reflectance calibration methods for airborne hyperspectral spectrometer data. Sensors 2009, 9, 794–813. doi:10.3390/s90200794. 22399939 PMC3280831

[62] Crowther B Radiometric Calibration of Multispectral Video Imagery. Ph.D. Thesis, Utah State University, Logan, UT, USA, 1992.

[63] Agisoft LL; St Petersburg R Agisoft Photoscan; Professional ed; 2014.

[64] Aboutalebi M; Torres-Rua AF; McKee M; Nieto H; Kustas WP; Prueger JH; McKee L; Alfieri JG; Hipps L; Coopmans C Assessment of Landsat Harmonized sUAS Reflectance Products Using Point Spread Function (PSF) on Vegetation Indices (VIs) and Evapotranspiration (ET) Using the Two-Source Energy Balance (TSEB) Model. AGU Fall Meet. Abstr 2018 Available online: https://ui.adsabs.harvard.edu/abs/2018AGUFM.H33I2193A/abstract (accessed on 15 December 2019).

[65] Torres-Rua A Vicarious Calibration of sUAS Microbolometer Temperature Imagery for Estimation of Radiometric Land Surface Temperature. Sensors 2017, 17, 1499. doi:10.3390/s17071499. PMC5539465 28672864

[66] Cook B; Corp LW; Nelson RF; Middleton EM; Morton DC; McCorkel JT; Masek JG; Ranson KJ; Ly V; Montesano PM NASA Goddard’s Lidar, Hyperspectral and Thermal (G-LiHT) airborne imager. Remote Sens 2013, 5, 4045–4066. doi:10.3390/rs5084045.

[67] Nieto H; Kustas WP; Torres-Rúa A; Alfieri JG; Gao F; Anderson MC; White WA; Song L; Alsina M.d.M.; Prueger JH; Evaluation of TSEB turbulent fluxes using different methods for the retrieval of soil and canopy component temperatures from UAV thermal and multispectral imagery. Irrig. Sci 2019, 37, 389–406. doi:10.1007/s00271-018-0585-9. PMC7192002 32355404

[68] Schotanus P; Nieuwstadt F; De Bruin H Temperature measurement with a sonic anemometer and its application to heat and moisture fluxes. Bound.-Layer Meteorol 1983, 26, 81–93. doi:10.1007/BF00164332.

[69] Liu H; Peters G; Foken T New equations for sonic temperature variance And buoyancy heat flux with an omnidirectional sonic anemometer. Bound.-Layer Meteorol 2001, 100, 459–468. doi:10.1023/A:1019207031397.

[70] Tanner CB; Thurtell GWT Anemoclinometer Measurements of Reynolds Stress and Heat Transport in the Atmospheric Surface Layer; Research and Development Technical Report ECOM 66-G22-F to the US Army Electronics Command; Dept. of Soil Science, Univ. of Wisconsin: Madison, WI, USA, 1969

[71] Massman W A simple method for estimating frequency response corrections for eddy covariance systems. Agric. For. Meteorol 2000, 104, 185–198. doi:10.1016/S0168-1923(00)00164-7.

[72] Webb EK; Pearman GI; Leuning R Correction of flux measurements for density effects due to heat and water vapour transfer. Q. J. R. Meteorol. Soc 1980, 106, 85–100. doi:10.1002/qj.49710644707.

[73] Foken T The Energy Balance Closure Problem: An Overview. Ecol. Appl 2008, 18, 1351–1367. doi:10.1890/06-0922.1. 18767615

[74] Oke T Boundary Layer Climates, 2nd ed.; Cambridge University Press: Cambridge, UK, 1987.

[75] Twine T; Kustas W; Norman J; Cook D; Houser P; Meyers T; Prueger J; Starks P; Wesely M Correcting eddy-covariance flux underestimates over a grassland. Agric. For. Meteorol 2000, 103, 279–300. doi:10.1016/S0168-1923(00)00123-4.

[76] Wilson K; Goldstein A; Falge E; Aubinet M; Baldocchi D; Berbigier P; Bernhofer C; Ceulemans R; Dolman H; Field C; Energy balance closure at FLUXNET sites. Agric. For. Meteorol 2002, 113, 223–243. doi:10.1016/S0168-1923(02)00109-0.

[77] Frank JM; Massman WJ; Ewers BE A Bayesian model to correct underestimated 3-D wind speeds from sonic anemometers increases turbulent components of the surface energy balance. Atmos. Meas. Tech 2016, 9, 5933–5953. doi:10.5194/amt-9-5933-2016.

[78] Frank JM; Massman WJ; Ewers BE Underestimates of sensible heat flux due to vertical velocity measurement errors in non-orthogonal sonic anemometers. Agric. For. Meteorol 2013, 171–172, 72–81. doi:10.1016/j.agrformet.2012.11.005.

[79] Horst TW; Semmer SR; Maclean G Correction of a Non-orthogonal, Three-Component Sonic Anemometer for Flow Distortion by Transducer Shadowing. Bound.-Layer Meteorol 2015, 155, 371–395. doi:10.1007/s10546-015-0010-3.

[80] Kochendorfer J; Meyers TP; Frank J; Massman WJ; Heuer MW How Well Can We Measure the Vertical Wind Speed? Implications for Fluxes of Energy and Mass. Bound.-Layer Meteorol 2012, 145, 383–398. doi:10.1007/s10546-012-9738-1.

[81] Vegetation Spectral-Structural Information eXtraction Algorithm (VSSIXA): Working with Point cloud and LiDAR https://github.com/Mahyarona/VSSIXA (accessed on 15 December 2019).

[82] Aboutalebi M; Allen LN; Torres-Rua AF; McKee M; Coopmans C Estimation of soil moisture at different soil levels using machine learning techniques and unmanned aerial vehicle (UAV) multispectral imagery In Proceedings of the Autonomous Air and Ground Sensing Systems for Agricultural Optimization and Phenotyping IV, Baltimore, MD, USA, 14–18 4 2019; Volume 11008. doi:10.1117/12.2519743.

[83] Schmidt M; Lipson H Distilling free-form natural laws from experimental data. Science 2009, 324, 81–85. 19342586 10.1126/science.1165893

[84] Schmidt M; Lipson H Eureqa (Version 0.98 beta) [Software]. 2014 Available online: www.nutonian.com (accessed on 15 December 2019).

[85] Kustas WP; Norman JM A two-source approach for estimating turbulent fluxes using multiple angle thermal infrared observations. Water Resour. Res 1997, 33, 1495–1508. doi:10.1029/97WR00704.

[86] Kustas WP; Norman JM Evaluation of soil and vegetation heat flux predictions using a simple two-source model with radiometric temperatures for partial canopy cover. Agric. For. Meteorol 1999, 94, 13–29. doi:10.1016/S0168-1923(99)00005-2.

[87] Campbell G; Norman J An Introduction to Environmental Biophysics; Modern Acoustics and Signal; Springer: New York, NY, USA, 2000.

[88] Willmott CJ; Matsuura K Advantages of the mean absolute error (MAE) over the root mean square error (RMSE) in assessing average model performance. Clim. Res 2005, 30, 79–82. doi:10.3354/cr030079.

[89] Despotovic M; Nedic V; Despotovic D; Cvetanovic S Evaluation of empirical models for predicting monthly mean horizontal diffuse solar radiation. Renew. Sustain. Energy Rev 2016, 56, 246–260. doi:10.1016/j.rser.2015.11.058.

[90] Li MF; Tang XP; Wu W; Liu HB General models for estimating daily global solar radiation for different solar radiation zones in mainland China. Energy Convers. Manag 2013, 70, 139–148. doi:10.1016/j.enconman.2013.03.004.

[91] Kljun N; Calanca P; Rotach MW; Schmid HP A simple two-dimensional parameterisation for Flux Footprint Prediction (FFP). Geosci. Model Dev 2015, 8, 3695–3713. doi:10.5194/gmd-8-3695-2015.

[92] Agam N; Kustas WP; Alfieri JG; Gao F; McKee LM; Prueger JH; Hipps LE Micro-scale spatial variability in soil heat flux (SHF) in a wine-grape vineyard. Irrig. Sci 2019, 37, 253–268. doi:10.1007/s00271-019-00634-6.

[93] Gao F; Anderson MC; Kustas WP; Houborg R Retrieving Leaf Area Index From Landsat Using MODIS LAI Products and Field Measurements. IEEE Geosci. Remote Sens. Lett 2014, 11, 773–777. doi:10.1109/LGRS.2013.2278782.

[94] Gao F; Kustas WP; Anderson MC A Data Mining Approach for Sharpening Thermal Satellite Imagery over Land. Remote Sens 2012, 4, 3287–3319. doi:10.3390/rs4113287.

[95] Nieto H; Kustas WP; Alfieri JG; Gao F; Hipps LE; Los S; Prueger JH; McKee LG; Anderson MC Impact of different within-canopy wind attenuation formulations on modelling sensible heat flux using TSEB. Irrig. Sci 2019, 37, 315–331. doi:10.1007/s00271-018-0611-y.

[96] Villagarcía L; Were A; Domingo F; García M; Alados-Arboledas L Estimation of soil boundary-layer resistance in sparse semiarid stands for evapotranspiration modelling. J. Hydrol 2007, 342, 173–183. doi:10.1016/j.jhydrol.2007.05.023.

[97] Andreu A; Kustas WP; Polo MJ; Carrara A; González-Dugo MP Modeling Surface Energy Fluxes over a Dehesa (Oak Savanna) Ecosystem Using a Thermal Based Two-Source Energy Balance Model (TSEB) I. Remote Sens 2018, 10, 567. doi:10.3390/rs10040567.

[98] Chávez JL; Gowda PH; Howell TA; Neale CMU; Copeland KS Estimating hourly crop ET using a two-source energy balance model and multispectral airborne imagery. Irrig. Sci 2009, 28, 79–91. doi:10.1007/s00271-009-0177-9.

[99] Kustas WP; Alfieri JG; Nieto H; Wilson TG; Gao F; Anderson MC Utility of the two-source energy balance (TSEB) model in vine and interrow flux partitioning over the growing season. Irrig. Sci 2019, 37, 375–388. doi:10.1007/s00271-018-0586-8.

